# scMaSigPro: differential expression analysis along single-cell trajectories

**DOI:** 10.1093/bioinformatics/btae443

**Published:** 2024-07-08

**Authors:** Priyansh Srivastava, Marta Benegas Coll, Stefan Götz, María José Nueda, Ana Conesa

**Affiliations:** BioBam Bioinformatics S.L., Valencia, 46024, Spain; Department of Computer Science, University of Valencia, Valencia, 46100, Spain; BioBam Bioinformatics S.L., Valencia, 46024, Spain; BioBam Bioinformatics S.L., Valencia, 46024, Spain; Mathematics Department, University of Alicante, Alicante, 03690, Spain; Institute for Integrative Systems Biology (I2SysBio), Consejo Superior de Investigaciones Cientıficas (CSIC), Paterna, 46980, Spain

## Abstract

**Motivation:**

Understanding the dynamics of gene expression across different cellular states is crucial for discerning the mechanisms underneath cellular differentiation. Genes that exhibit variation in mean expression as a function of *Pseudotime* and between branching trajectories are expected to govern cell fate decisions. We introduce *scMaSigPro*, a method for the identification of differential gene expression patterns along *Pseudotime* and branching paths simultaneously.

**Results:**

We assessed the performance of *scMaSigPro* using synthetic and public datasets. Our evaluation shows that *scMaSigPro* outperforms existing methods in controlling the False Positive Rate and is computationally efficient.

**Availability and implementation:**

*scMaSigPro* is available as a free R package (version 4.0 or higher) under the GPL(≥2) license on GitHub at ‘github.com/BioBam/scMaSigPro’ and archived with version 0.03 on Zenodo at ‘zenodo.org/records/12568922’.

## 1 Introduction

Single-cell RNA-seq (scRNA-Seq) datasets, particularly those featuring cell differentiation and transition experiments, greatly benefit from Trajectory Inference (TI) analysis. TI method reveals the dynamics of cellular processes by assigning *Pseudotime* to individual cells (Trapnell *et al.* 2014). *Pseudotime* allocation facilitates the ordering of cells along the continuum of cellular processes and delineates the course of cell lineages as specific *paths* or *branches* within a scRNA-Seq dataset (Deconinck *et al.* 2021). By associating *Pseudotime* to gene expression values, transcriptional profiles across cell states can be delineated. Genes that show significant variation of their mean expression in *Pseudotime* along a specific branch of a trajectory are expected to play crucial roles in cell fate decisions. Identifying these genes is key to understanding the mechanisms of cell differentiation (Song and Li 2021).

Several methods, including tradeSeq, PseudotimeDE, and Monocle3, have been developed to detect Differentially Expressed (DE) genes along the inferred *Pseudotime* (Cao *et al.* 2019, Van Den Berge *et al.* 2020, Song and Li 2021). However, these methods face challenges. Some are limited to detecting linear patterns, while others struggle with modelling gene expression along branching paths and *Pseudotime* simultaneously (Song and Li 2021). Additionally, Generalized Additive Models (GAMs), commonly employed by these methods, are prone to overfitting and pose interpretability challenges due to their black-box nature. Moreover, as the dimensionality of the dataset increases, the computational performance of GAMs can significantly decline. Previous studies have highlighted that zero-inflated (ZI) versions of GAMs and Generalized Linear Models (GLMs) often result in a power loss, leading to high False Negative Rates (FNR) (Silverman *et al.* 2020).

In response to these challenges, we introduce *scMaSigPro*, an adaptation of *maSigPro* (Conesa *et al.* 2006, Nueda *et al.* 2014), a method initially developed for serial analysis of transcriptomics data, to the analysis of scRNA-seq trajectory data. *scMaSigPro* is specifically designed to detect genes that change their expression in *Pseudotime* and between branching paths, thereby identifying genes critical to cell-fate decisions.

## 2 Materials and methods

*maSigPro* utilizes Polynomial Generalized Linear models (Poly-GLM) to model gene expression over time across one or multiple experimental groups, identifying genes that exhibit differential expression profiles over time. However, the direct application of *maSigPro* to scRNA-seq data is challenging due to the higher levels of intrinsic noise from stochastic gene expression, sparsity resulting from technical dropouts, and the inherently high dimensionality of the data.

**Figure 1.**
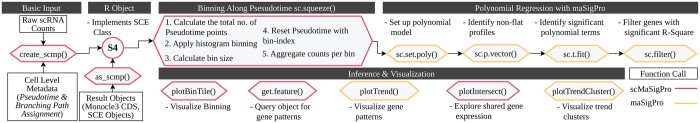
The workflow of *scMaSigPro* begins with the creation of an S4 object, followed by binning, model fitting, and visualization. Functions in yellow are adapted from *maSigPro* while functions in pink are newly implemented in *scMaSigPro*.

To tailor the *maSigPro* algorithm for scRNA-seq trajectory analysis, three major modifications were made: (i) a binning strategy was introduced to create pseudo-bulk or binned *Pseudotime* points to compensate for the low signal-to-noise ratio of the scRNA-seq counts. (ii) An equalizing method was employed to manage the uneven distribution of cells along a branching path. (iii) The Poly-GLM model was enhanced by incorporating size factors to adjust for variations in bin sizes. The majority of *maSigPro* functions have been modified to accommodate the new ‘scMaSigPro S4’ Class, enabling parallel computation during model fitting. Additionally, new functions have been introduced to the package to facilitate the implementation of these adaptations (Fig. 1, Supplementary Fig. S1).

### 2.1 Binning along *pseudotime*

*Pseudotime* values serve as indicators of the cells’ positions within a dynamic biological process. Therefore, grouping cells based on their *Pseudotime* values effectively clusters cells that are in similar states into the same category or bin (Supplementary Fig. S2).

In the *scMaSigPro* workflow, the original *Pseudotime* values, which are continuous, are discretized into *Binned Pseudotime* value, which is the ordinal index of the bins capturing cells with similar states.

The binning along the *Pseudotime* is carried out using the ‘sc.squeeze()’ function, and the determination of the bins (size and numbers) is guided by one of the histogram binning strategies outlined in the Supplementary Material, Supplementary Section S1.1. For clarity, we demonstrate how *scMaSigPro* conducts binning using the ‘Sturges’ Rule.

Let *N* be the number of inferred *Pseudotime* values, collected in a vector $T_{\mathrm{pseudotime}}=(t_{1},t_{2},t_{3},t_{4},\ldots ,t_{N})$.

The number of bins is (*B_number_*) is calculated by $B_{\mathrm{number}}=([\log2(N)+1])\times k$, where k∈[0.3,∞] is a drop-factor, and [log2(N)+1] is the ‘Sturges’ formula.

The size of each bin, *B_size_*, is then defined by:

$$B_{\mathrm{size}}=\frac{\max(T_{\mathrm{pseudotime}})-\min(T_{\mathrm{pseudotime}})}{B_{\mathrm{number}}}$$

After the determination of the *B_size_*, *scMaSigPro* partitions the entire range of the original *Pseudotime* values and assigns an ordinal index to the bins. The bins are structured as left-closed and right-open intervals, except for the last bin, which is closed on both ends.

Finally, *scMaSigPro* aggregates the cell counts of each gene within each bin, resulting in a pseudo-bulk count per bin. This process enhances the signal-to-noise ratio. Binning is carried out separately for each branching path to maintain the distinctions in cell fate and to create expression profiles compatible with *maSigPro* analysis.

### 2.2 Equalizing heterogeneous cell distributions

Cell states can transition at different velocities or be captured variably in the scRNA-seq dataset, leading to an uneven distribution of cells along the differentiation trajectory. Consequently, the number of cells in each bin may substantially differ, affecting the uniformity of counts aggregated across *Binned Pseudotime Points* and challenging the assumptions of homoscedasticity.

To address this issue, *scMaSigPro* incorporates a bin equalization step, enabled by default with the split_bin parameter set to TRUE. *scMaSigPro* calculates the mean (*μ*) and standard deviation (*σ*) of the sizes of all bins per path to establish maximum (μ+σ) and minimum (μ−σ) allowable bin sizes. Bins larger than the maximum size are divided, and those smaller than the minimum size can be optionally removed, ensuring uniform bin sizes. The process is repeated until all bins conform to the established size criteria.

### 2.3 Polynomial generalized linear model

After obtaining the pseudo-bulk counts for each *Binned Pseudotime Point* along every branching path, *scMaSigPro* establishes the polynomial model by assigning dummy variables to each branch, following the approach of the original *maSigPro* method (Conesa *et al.* 2006) for the Generalized Linear Model (GLM) described in Nueda *et al.* (2014).

For clarity, let us consider two developmental trajectories stemming from common progenitor cells (*Prog_Cells_*), which diverge into $\mathrm{Pro}g_{\mathrm{Cells}}\to \mathrm{Cel}l_{A}$ and $\mathrm{Pro}g_{\mathrm{Cells}}\to \mathrm{Cel}l_{B}$. Each cell’s trajectory is indicated by a binary variable *j*, where *j *=* *0 corresponds to the path leading to *Cell_A_* and *j *=* *1 to *Cell_B_*. After applying the binning strategy discussed in the previous section over the inferred *Pseudotime* of each branching path, the binned *Pseudotime* will be given by $I\in \{1,\ldots ,B_{\mathrm{number}}\}$ per branching path. The variable *Y_ij_*, representing the observed counts of gene *i* in bin *j*, is modelled as follows:

(1) $$g(\mu_{ij})=\beta_{0}+\beta_{1}t_{i}+\beta_{2}t_{i}^{2}+\beta_{3}z_{j}+\beta_{4}t_{i}z_{j}+\beta_{5}t_{i}^{2}z_{j}+\mathrm{Offse}t_{ij}$$

Here, g(.) is the ‘*link function*’ that characterizes the GLM (McCullagh and Nelder 2019), effectively transforming the linear predictor into the appropriate scale for the response variable, which is the pseudo-bulk count in this model. The term $\mu_{ij}=E(Y_{ij})$ represents the expected mean of the counts, conditioned on the *binned Pseudotime* (*t_i_*) and the binary variable *z_j_*.

The default model assumes a Negative Binomial distribution, $Y_{ij}∼NB(\mu_{ij},\theta )$, and sets the ‘*link function*’ to the logarithmic function, $g(\mu_{ij})=\log(\mu_{ij})$, although this can be changed by the user (e.g. to ‘identity’) for other data distributions. The logarithmic link function ensures that the expected counts (*μ_ij_*) are positive, and it is particularly appropriate for the overdispersed count data typically found in scRNA-Seq experiments. The parameter *θ* represents the dispersion parameter of the Negative Binomial distribution, addressing the overdispersion commonly seen in this type of data.

Finally, unlike *maSigPro* that relies on normalized expression values, *scMaSigPro* incorporates an offset value to the Poly-GLM model to adjust for disparities in total read counts per bin. This offset is calculated as the logarithm of size factors for each bin (Supplementary Material, Supplementary Section S1.3).

### 2.4 Workflow

*scMaSigPro* implements a two-step strategy similar to the *maSigPro* method, which consists of first identifying genes with dynamic profiles and then applying stepwise regression to select the best model for each gene (Conesa *et al.* 2006, Nueda *et al.* 2014).

Initially, *scMaSigPro* fits the full polynomial model for each gene, as outlined in equation-1, using the ‘sc.p.vector()’ function. This model incorporates all polynomial terms to capture the full range of potential gene expression dynamics. Subsequently, the adequacy of each gene’s full model is evaluated against a simpler intercept-only model via hypothesis testing based on the log-likelihood ratio statistic as it is explained in Nueda *et al.* (2014). Only genes whose full models meet the significance threshold (default is 0.05) proceed to the next stage.

In the next stage, stepwise regression is employed through the ‘sc.t.fit()’ function to refine the models that passed the initial significance filter. This process iteratively eliminates nonsignificant polynomial terms and returns a model that best fits the expression profile of each gene.

In this second stage, the goodness of fit, *R*^2^, of each optimized gene model is computed. This parameter is used to select genes with well-defined expression trends. In Linear Models, *R*^2^ is defined from the residual sum of squares, while in Generalized Linear Models, the goodness of fit is evaluated as the percentage of deviance explained by the model (Nueda *et al.* 2014).

Lastly, the list of significant genes is selected based on whether they have a relatively high *R*^2^ associated with its model (Nueda *et al.* 2014). The selected genes can be subject to clustering, effectively revealing trends in *Pseudotime*.

### 2.5 Evaluation with synthetic data

We used Splatter (Zappia *et al.* 2017) to generate synthetic datasets, including 2000 genes and 3000 cells, featuring a bifurcation topology (Supplementary Fig. S3).

Splatter mimics differential expression by simulating fold changes between the start and the end of the cell trajectory. Let the base expression of gene *g_i_* be denoted as ***α***_*i*_ in branching path *path_j_*, and the expression of *g_i_* at the end of *path_j_* be ***γ***_*i*_. The effective change in expression, denoted as ***δ***_*i*_, across the simulated *Pseudotime* can be obtained by calculating the difference between ***α***_*i*_ and ***γ***_*i*_. The expression for ***δ***_*i*_ is given by:

(2) $$\delta_{i}=\gamma_{i}-\alpha_{i}$$

**Figure 2.**
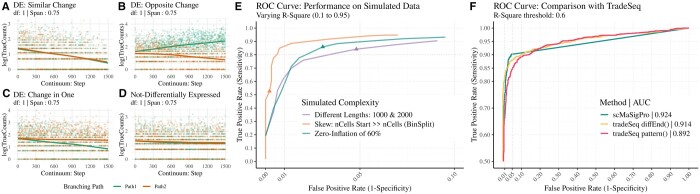
(A–D) Simulated ground truth expression of genes and corresponding patterns. (A–C) Differentially Expressed Genes in *Pseudotime*. (A) A simulated gene showing similar expression change in *Pseudotime* in both the branching paths. (B) A simulated gene showing dissimilar expression change in *Pseudotime* in both the branching paths. (C) A simulated gene showing expression changes only in one path in *Pseudotime*. (D) No difference along the *Pseudotime* or among the branching paths is considered Not Differentially Expressed. (E) ROC curves for three synthetic datasets show the performance at varying *R*^2^ values. The triangles represent values at *R*^2^ of 0.6. (F) Performance comparison between *scMaSigPro* and tradeSeq, the former exhibits tighter FPR control with a slight increase in TPR. Genes that do not meet the *scMaSigPro* selection criteria (*P*-value ≤ 0.05 and *R*^2^≥ 0.6) are assigned a *P*-value of 1 during iCOBRA evaluation. This assignment leads to a flattened ROC curve when the False Positive Rate exceeds 0.05.

If the value of ***δ***_*i*_ is not equal to 0, then gene *g_i_* is called to be differentially expressed in *Pseudotime* along pathway *path_j_* (Fig. 2A–C). If the value is 0, we consider the gene not differentially expressed (Fig. 2D).

To closely replicate real scRNA-seq data, simulation parameters were estimated from the raw counts reported in Setty *et al.* (2019). Specifically, we estimated the inherent characteristics of the real dataset such as *library size*, *drouputs* and *biological coefficient of variation* (BCV). Finally, we used Splatter’s splatSimulate(method = ‘paths’) function to simulate a differentiation process. This base simulation displayed approximately 38% sparsity, with each branching path comprising around 1500 cells evenly distributed. A total of 550 genes were simulated to be DE along the trajectory (Supplementary Material, Supplementary Section S2).

To assess the influence of dropout events, four synthetic datasets with increasing sparsity levels were generated, ranging from 60% to approximately 90% in 10% increments (Supplementary Fig. S4A–D). We adjusted the dropout.shape parameter in the ‘splatSimulate()’ function to increase sparsity levels in combination with the base 38% sparsity learned from the data. To evaluate scenarios where certain cell states are captured more frequently than others, resulting in uneven distribution of cells across the *Pseudotime* (or *skewed cell distributions*), we created four more datasets (Supplementary Fig. S5A–D). In Splatter, the degree of skewness is regulated by the path.skew parameter (default at 0.5), with settings near 0 producing paths where cells predominantly accumulate at the end (indicating an abundance of differentiated cells), and settings close to 1 biasing cell accumulation towards the start (indicating a predominance of stem cells). Two datasets were simulated with strong left skewness (path.skew** **=** **0.9 & 1) and two with strong right skewness (path.skew** **=** **0 & 0.1).

In our final set of simulations, we generated four datasets to represent variable developmental times, which can result in branches of differing lengths (Supplementary Fig. S6A–D). Specifically, a quick transition of a cell to its end state is characterized by a shorter *Pseudotime* range, while a more gradual transition results in longer *Pseudotime* ranges. To model such scenarios, we adjusted the path.nSteps parameter, which specifies the number of steps between the start and endpoints of a path.

For all simulated datasets, full models (i.e. all polynomial terms with equation-1) were initially computed using a cubic polynomial degree to capture potential nonlinear expression trends along *Pseudotime* with ‘sc.p.vector()’ function. Subsequently, stepwise regression was applied to selected significant full models (*P*-value < 0.05) using ‘sc.t.fit()’ function. The *R*^2^ of the final significant models containing only significant terms were obtained.

We generated Operating Characteristic (ROC) curves by varying the *R*^2^ threshold from 0.1 to 0.95 in increments of 0.05. Genes were classified as DE if the model’s *R*^2^ exceeded the threshold and not DE otherwise. Performance metrics such as True Positive Rate (TPR), False Positive Rate (FPR), Accuracy, and F1 Score were calculated based on these classifications.

### 2.6 Comparison with tradeSeq

We compared the performance of *scMaSigPro* with tradeSeq (Van Den Berge *et al.* 2020), a negative binomial GAM framework, that fits cubic splines to each branching path on the same *K* cubic basis functions. In addition, tradeSeq includes covariates that represent each cell and offsets in the model (Supplementary Material, Supplementary Section S3). Apart from the differences between *scMaSigPro* and tradeSeq fitted models, they differ in the gene selection criteria. While tradeSeq prioritizes genes based exclusively on their *P*-values, *scMaSigPro* also incorporates a measure of the quality of the model, the percentage of deviance explained by the GLM, i.e. *R*^2^.

To compare and evaluate the difference between the tradeSeq and *scMaSigPro*, we simulated another dataset with 3000 cells and 2000 genes (Supplementary Fig. S7). We simulated all the stated complexities in the previous section, i.e. sparsity, skewness and different branch lengths, in the same dataset. Globally, 30% of the genes were simulated to be DE.

In accordance with the guidelines outlined in Van Den Berge *et al.* (2020), we first normalized the raw simulated counts using the ‘FQ normalization script’ available from the tradeSeq package. Subsequently, we used the ‘evaluateK()’ function from tradeSeq to determine the optimal number of knots, ranging from 3 to 15 (Supplementary Fig. S8). This analysis identified five as the optimal number, which we then applied in the ‘fitGAM()’ function, maintaining all other parameters at their default settings. After completing the model fitting, we employed the ‘patternTest()’ and ‘diffEndTest()’ function to generate lists of DE genes together with their corresponding *P*-values.

The comparative analysis was conducted using the iCOBRA framework (Soneson and Robinson 2016, Supplementary Material, Supplementary Section S3.2). To make *scMaSigPro* results compatible with the iCOBRA package, nonsignificant genes [*P*-value ≥ 0.05 after ‘sc.p.vector()’ function] and gene with low *R*^2^*(R*^2^≤ 0.6 after ‘sc.t.fit()’ function) were assigned a *P*-value of 1. This allowed the evaluation of gene rankings and filtered genes with the iCOBRA framework.

### 2.7 Computational runtime

Taking advantage of the parallel processing capabilities offered by both packages, we assessed their computational runtime using 1 and 8 CPU cores on a laptop with Core i7-9700K clocked at 2.90 GHz with 8 CPU cores (4 + 4 virtual). We used R’s ‘microbenchmark’ package to evaluate the wall clock time taken by individual functions. For tradeSeq, ‘fitGAM()’ and ‘patternTest()’ where monitored, while *‘*sc.p.vector() and ‘sc.t.fit()’ were evaluated in *scMaSigPro*. For any of the comparisons, we did not consider the additional time that might be used to estimate the optimal number of knots with the ‘evaluatek()’ function.

We first evaluated a sub-sample of 1500 cells and 1000 genes from the simulated dataset, which was used for comparison. Next, we evaluated a bigger simulated dataset of 6000 cells, each branch having 3000 cells ten times. Unfortunately, we could not compare tradeSeq with >6000 cells with 8 CPU cores on a laptop with 32 GB memory. Performing such computation with tradeSeq produces large memory overheads, making the R session crash. However, *scMaSigpro* is scalable and can efficiently use memory. Thus, to evaluate further, we simulate 4 datasets with an increasing number of cells, up to 10 000 cells per branching path. In this evaluation, we considered the ‘sc.p.vector()’ and ‘sc.t.fit()’ functions individually and evaluated them ten times.

### 2.8 Public data

We examined the CD34+ enriched stem/progenitor cells (HSPCs) dataset from Setty *et al.* (2019), which includes differentiating HSPCs from three healthy donors.

The raw FastQ files were downloaded from the European Nucleotide Archive (ENA), having project accession as PRJEB37166. Cell Ranger v7 was used to align the reads to the genome (Zheng *et al.* 2017). Specifically, we utilized the ‘GRCh38.primary_assembly’ from GENCODE, along with the annotation GTF ‘gencode.v43.annotation.filtered.gtf’. The Cell Ranger ‘count’ was run independently for each donor to generate three digital feature barcode matrices.

We used Seurat (version 5.0.1) for quality control and filtering (Hao *et al.* 2023) (Supplementary Table S3). As per methods in Setty *et al.* (2019), we also removed the cell cycle effects from each donor. Specifically, we used the reference ‘Human Bone marrow’ from Azimuth References, which consisted of 297 627 bone marrow cells from 39 donors and three different studies performed with 10x Genomics v2 chemistry to match with Setty *et al.* (2019) (Hao *et al.* 2023).

Next, we sub-sampled each dataset to exclude cell types typically not originating from the bone marrow, such as stromal cells. Using Monocle3, trajectories were inferred with HSCs designated as the starting cells. Specifically, the trajectory graph was learned using default parameters, except for the ‘close loop’, ‘use partition’, and ‘prune graph’, which were set to FALSE (Supplementary Fig. S15). For each of the donors, a distinct set of branching paths was analyzed. The results for Donor-1, showcasing Prog Megakaryocytes (ProgMk) and Early Erythrocytes (EarlyE) originating from Erythro-Myeloid Progenitors (EMPs/MEPs), are presented here. Results for Donor-2/3 are detailed in the Supplementary Material, Supplementary Section S4.

The inferred *Pseudotime* and the definitive assignment of each cell to its corresponding branch served as the input for *scMaSigPro*. We applied cubic poly-GLM to accurately model the nonlinear gene expression patterns observed as MEPs differentiate into ProgMk or EarlyE. Significant genes expected to mediate differentiation towards ProgMk were selected by *scMaSigPro* at an *R*^2^ of 0.7. The resulting gene list was used for clustering and a Gene Ontology (GO) enrichment analysis, using all detected genes as background.

## 3 Results

### 3.1 Synthetic data

Synthetic data was used to assess the performance of *scMaSigPro* across various scenarios with different levels of sparsity, cell distribution skewness, and branch lengths.

*Zero-Inflation: scMaSigPro* demonstrated a high True Positive Rate (TPR) close to 90% and a False Positive Rate (FPR) below 5% when Zero-Inflation (ZI) levels were set to 60% and 70% (Fig. 2E) at an *R*^2^ range of 0.45–0.6. A minor reduction in TPR was noted as ZI increased to 80%, while extreme ZI levels significantly affected the detection of true positives (Supplementary Fig. S4E–H). Nonetheless, the FPR consistently stayed below the 0.05 threshold in all cases. These findings underscore the robustness *scMaSigPro* to varying degrees of sparsity and its effectiveness in minimizing false discoveries under realistic data scenarios. For datasets with >80% ZI, data imputation is advisable prior to *scMaSigPro* analysis.

*Skewness: scMaSigPro* effectively controlled FPR in datasets with high skewness while maintaining the TPR around 80% (Fig. 2E). The direction of skewness influenced the FPR (Supplementary Fig. S5E–L), which significantly dropped upon activating the split_bins option, which splits disproportionately large bins into smaller ones to improve performance (Fig. 2E).

*Unequal lengths of branching paths:* In cases of branching paths were of similar length, *scMaSigPro* showed optimal performance with the TPR approaching 90% and FPR below 5% (Fig. 2E). In scenarios where the lengths of branching paths significantly differ (i.e. >2-folds), the TPR decreased to 50% at an *R*^2^ threshold of 0.6 (Supplementary Fig. S6E–H), while the FPR was maintained between 5–10%. Given these findings, subsetting the dataset to ensure more uniformity in trajectory lengths can potentially enhance the *scMaSigPro* accuracy, mitigating the elevated FPR.

### 3.2 Comparison with tradeSeq

The ‘diffEndTest()’ function from tradeSeq, which identifies genes that show differential patterns towards the end of *Pseudotime* and between the branching paths, performed comparably to *scMaSigPro*, while the ‘patternTest()’ function from tradeSeq that detects differential patterns across and between the branching path *Pseudotime* resulted in a higher number of False Positives (FPs) (Fig. 2F). In our comparative analysis, *scMaSigPro* demonstrated better performance over tradeSeq in controlling the FPR; however, the TPR was only marginally better (Fig. 2F).

We investigated the characteristics of False negative (FN) genes, i.e. those incorrectly identified as not DE by each method. We observed that *scMaSigPro* struggled to identify genes with low fold changes, particularly when a gene is DE in only one of the branching paths and exhibits a stable expression pattern in the other (Supplementary Figs S9 and S10). Such patterns were usually detected by the tradeSeq’s ‘diffEndTest()’ and ‘patternTest()’ functions.

### 3.3 Computational runtime

When utilizing a single CPU, *scMaSigPro* proved to be four times faster, resulting in a lower carbon footprint (30.53 mg CO2e) compared to tradeSeq (122.14 mg CO2e) (Lannelongue *et al.* 2021, Supplementary Fig. S11, Supplementary Table S1). This performance advantage increases with the use of 8 CPUs, where *scMaSigPro* is five times faster than tradeSeq. Also, the performance was consistent on the larger dataset of 6000 cells (Supplementary Fig. S12).

Evaluation of the individual function of *scMaSigPro*, with datasets up to 10 000 cells per branch, shows a linear increase in the runtime with an increasing number of cells (Supplementary Fig. S13). The ‘sc.p.vector()’ function was found to be considerably faster than the ‘sc.t.fit()’ as a result of global model fitting. Although ‘sc.t.fit()’ function consumes more time due to stepwise regression, it greatly benefits from more cores, which allow faster evaluations, simultaneously keeping the memory overhead minimal, which is typically challenging in R.

### 3.4 Public data

The cell types were annotated using Azimuth References (Bone Marrow) based on the cell-specific markers listed in the Supplementary Table S2. The top 6000 Hypervariable genes were used for the analysis, including UMAP visualization (Supplementary Fig. S14, Supplementary Table S3). Next, after pre-processing the data with Seurat, we transferred the UMAP embedding to Monocle3 for trajectory inference and selected different branching paths per donor for *scMaSigPro* input (Supplementary Fig. S15).

The dataset from Donor-1 (Age 35, Male) contained 195 cells and 6000 genes. *scMaSigPro* divided Erythrocyte-Lineage into six bins and ProgMk-Lineage into three bins, averaging 19 and 27 cells per bin, respectively (Supplementary Fig. S16). Using a cubic polynomial model, Erythrocyte-Lineage as a reference and a *R*^2^ threshold of 0.7, *scMaSigPro* identified 300 features exhibiting significant and consistent nonflat expression trends (Supplementary Table S4). Among these, 278 genes showed significant change in expression between ProgMk-Lineage and Erythrocyte-Lineage with *Pseudotime* and, 22 showed significant change in expression with *Pseudotime*, but without significant differences between the lineages. Subsequently, clustering analysis of 278 genes using the ‘sc.cluster.trend()’ function resulted in six clusters (Supplementary Fig. S17).

**Figure 3.**
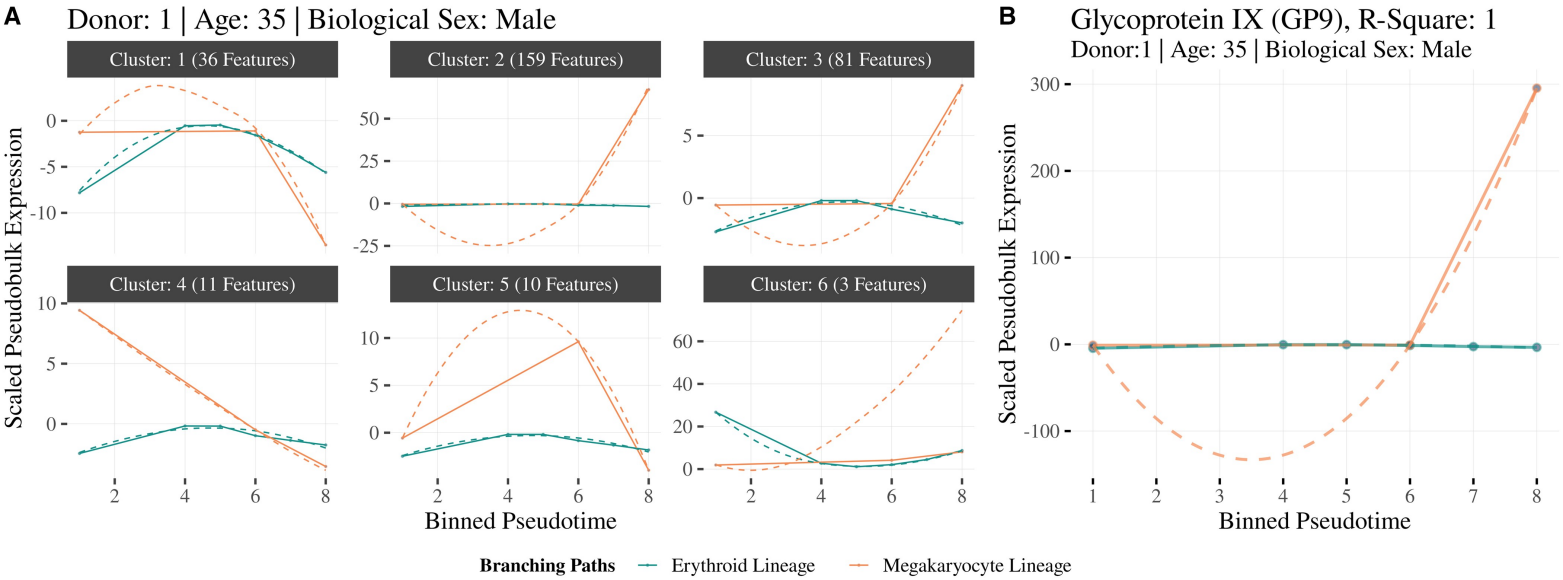
Analysis of genes with differential trajectories in the HSPC dataset. (A) Clustering of genes identified as differential by *scMaSigPro* for Donor-1. Clusters 2 and 3 (240 genes) showed an upward trend in the Megakaryocyte Lineage. (B) *scMaSigPro* models of GP9 expression reveal increased expression as MEPs differentiate into ProgMk and stable expression within the Erythrocyte Lineage.

Genes in Cluster 2 (159 Genes) and Cluster 3 (81 Genes) showed an increasing profile in ProgMk-Lineage (Figure 3A) . This gene list of 240 genes was enriched in terms such as ‘response to wound healing’, ‘blood coagulation’, and ‘platelet activation’, pointing towards the differentiation process of the HSPCs to Megakaryocyte Lineage resulting in platelets (Supplementary Fig. S18). Manual inspection of the gene list confirmed the presence of known markers such as LTBP1, ARHGAP6, GP9, SPX, SELP, RBPMS2 and WFDC1 (Supplementary Fig. S19). Glycoprotein IX (GP9) is critical in platelet adhesion and aggregation, particularly under high-shear stress conditions. Further results for additional donors are outlined in the Supplementary Materials (Fig. 3B).

## 4 Discussion

*scMaSigPro* unlocks the utilization of poly-GLM models for differential trajectory analysis of scRNA-seq data by applying a *Pseudotime* binning approach that preserves the original cell order along the *Pseudotime* while reducing data sparsity and heterogeneity. Previous studies have shown that pseudo-bulk methods overperform approaches that modelled cells individually (Murphy and Skene 2022). Our benchmarks confirm that *Pseudotime* binning significantly enhances the sensitivity and yields a consistently high TPR over a wide range of realistic data scenarios. For datasets with extreme ZI (over 85%), we recommend adjusting the drop factor (‘*k*’) to increase compression, yielding fewer but more balanced bins per path.

The ‘sc.squeeze()’ function in *scMaSigPro* provides various adjustable parameters that can be tailored according to the range of the *Pseudotime* values. For example, Rice’s rule is suitable when *Pseudotime* values span a broad range. Alternatively, Doane’s Binning formula is preferred for nonnormally distributed *Pseudotime* values as it accounts for skewness. The drop_tails parameter is useful when the user wants to focus only on the comparable *Pseudotime* segments of the bifurcating trajectories. Activating this option will eliminate any additional bins that extend beyond the *Pseudotime* bin of the shortest path. The prune_bin parameter removes bins not meeting the minimum size criteria (μ−σ).

Parameters in the ‘sc.p.vector()’ and ‘sc.t.fit()’ functions offer flexibility for adapting to the characteristics of the single-cell data. For instance, in our analysis of the HSPC public dataset, cell-cycle effects were removed, and counts were transformed into continuous values. In such cases, the *link function* can be set to be a Gaussian distribution. Similarly, if counts are already normalized, users may opt to disable the incorporation of offsets in the GLM. Collectively, the functions ‘sc.squeeze()’ (new to *scMaSigPro*), ‘sc.pvector()’ and ‘sc.t.fit()’ (adapted from *maSigPro*), provides comprehensive control over the model fitting process.

*scMaSigPro* is therefore suited for diverse topologies and cell state compositions typical of cell differentiation scRNA-seq experiments. Compared to methods based on GAMs, *scMaSigPro* improves precision and significantly reduces computational time, representing a sustainable alternative to the processing of large-scale datasets.

In conclusion, *scMaSigPro* efficiently identifies DE genes along branching paths and the *Pseudotime*, controlling the FPR effectively. Its compatibility with existing R packages, such as Monocle3, enhances its integration in scRNA-seq analysis workflows. Future developments may include an extension to spatial transcriptomics and changes in cell communication during cellular dynamic processes.

## Supplementary Material

btae443_Supplementary_Data

## Data Availability

*scMaSigPro* is available as a free R package (version 4.0 or higher) on GitHub at 'github.com/BioBam/scMaSigPro' and archived with version 0.03 on Zenodo at 'zenodo.org/records/12568922'.
