# Supplementary material for: scMaSigPro: differential expression analysis along single-cell trajectories
Source: Bioinformatics. 2024 Jul 8;40(7):btae443. doi: 10.1093/bioinformatics/btae443 (PMC11269465; doi:10.1093/bioinformatics/btae443)
Supplement: btae443_Supplementary_Data [file btae443_supplementary_data.zip › Supplementary_Material.pdf]

scMaSigPro: Differential Expression Analysis along Single-Cell Trajectories
SUPPLEMENTARY MATERIAL

Priyansh Srivastava[1,2], Marta Benegas Coll[1], Stefan Götz[1], María José Nueda[3], and Ana Conesa[4]

[1]BioBam Bioinformatics S.L., Valencia, Spain
[2]Department of Computer Science, University of Valencia, Spain
[3]Mathematics Department, University of Alicante, Spain
[4]Institute for Integrative Systems Biology (I^{2}SysBio), Consejo Superior de Investigaciones Científicas (CSIC), Valencia, Spain

July 2024

Contents

1 Pseudotime Binning **2**
1.1 Histogram Binning Methods 2
1.2 Effect of Binning 3
1.3 Addition of offsets 3

2 Benchmarks **4**
2.1 Bifurcating Paths and Base Simulation 4
2.2 Varying levels of Zero-Inflation/Sparsity 5
2.3 Varying capture bias of cells (Skewness) 6
2.4 Unequal Length of Branching Paths 7

3 Comparison with tradeSeq **8**
3.1 Simulation of Data 8
3.2 Evaluation with iCobra 9
3.3 False Negatives 9
3.4 Evaluation of computational running time and scalability 10
3.4.1 Environmental Impact of Algorithm 10

4 Analysis of Public Data **12**
4.1 Data Preparation with Seurat 12
4.2 Cell Type Annotation & Sub-Sampling 12
4.3 Trajectory Inference 12
4.4 scMaSigPro Analysis 12
4.4.1 Setting Polynomial GLM 12
4.4.2 Model Fitting and Selection of Genes 14
4.4.3 Clustering Gene Expression Trends 15

5 Additional Information **19**

1 Pseudotime Binning

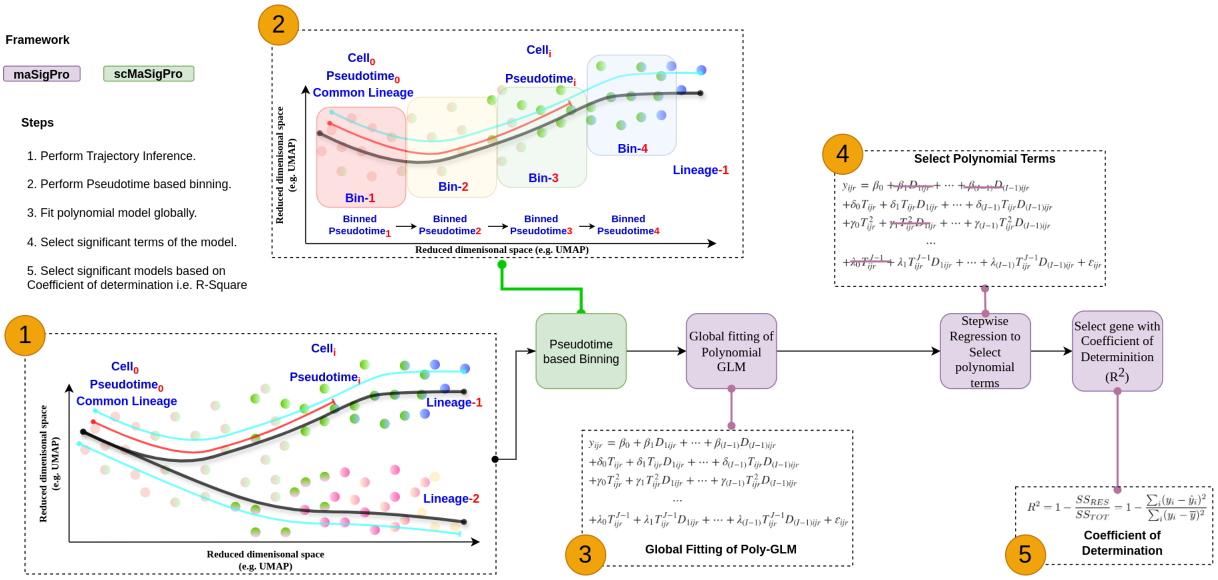

Supplementary Figure 1: Summary of the *scMaSigPro* workflow including the binning process. The binning is performed before the model fitting. The binning procedure replaces the original *Pseudotime* with the ordinal index of the bin representing the temporal dimension.

1.1 Histogram Binning Methods

- Square Root Formula: Default procedure applied to uniformly distributed *Pseudotime* values.

$$B = [(\sqrt{N}) * k]$$

- Sturges Rule: (Default Method) Widely applied in normally distributed *Pseudotime* data.

$$B = [(log_2(N) + 1) * k]$$

- Rice-Rule: Generates more bins and is suitable for data sets with long-range *Pseudotime* values.

$$B = [(2N^{1/3}) * k]$$

- Doane's Formula: Effective for non-normal distributions as it incorporates skewness into the calculation.

$$B = [(1 + \log_2(N) + \log_2\left(1 + \frac{|g_1|}{\sigma_{g_1}}\right)) * k]$$

  where g_1 is the estimated 3rd-moment-skewness of the distribution and $\sigma_{g_1} = \sqrt{\frac{6(N-2)}{(N+1)(N+3)}}$

- Scott Normal: Optimal for normally distributed *Pseudotime* values. It adjusts the bin width/size according to the variation in the *Pseudotime* values.

$$B = [(\frac{3.49\sigma'}{\sqrt{N}}) * k]$$

  where $3.49\sigma'$ is the sample standard deviation.

- Freedman Diaconis: It uses the interquartile range (IQR) to determine bin width, making it robust against outliers and skewed values. It's particularly useful for *Pseudotime* values with outliers or non-normal distribution.

$$B = [(2\frac{IQR(T)}{\sqrt[3]{N}}) * k]$$

  Where IQR(T) is the IQR of inferred *Pseudotime*.

Once the number of bins is obtained using one of the methods section-1.1, the continuous *Pseudotime* is converted to discrete units using R-Package 'entropy' with 'entropy()' function (Jean Hausser and Korbinian Strimmer, 2021).

1.2 Effect of Binning

To ensure that during the binning procedure, the original gene expression trend along *Pseudotime* is not changed, we obtained the 'LOESS' fit along the binned *Pseudotime* and the original inferred *Pseudotime*. Supplementary Figure 2 shows that the original trend is preserved.

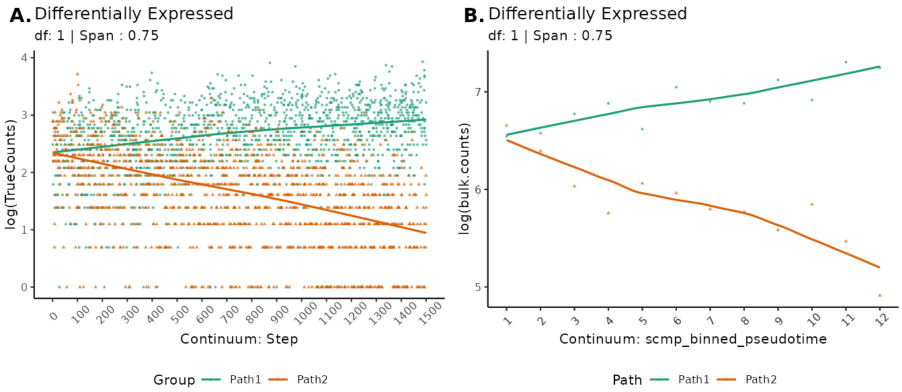

Supplementary Figure 2: Comparison of a simulated gene '`Gene 691`' before and after binning. Lines represent trends of the gene in two distinct paths with 'LOESS' fit. (A) Expression of the gene plotted against Steps (Pseudotime-Like variable 2.1) using True-counts after the simulation. (B) Expression of the same gene after binning with *scMaSigPro* on the simulated counts. After the binning, the trend of the gene across the continuum is preserved.

1.3 Addition of offsets

scMaSigPro includes an offset term in the poly-GLM model to account for the library size differences among bins. The offset term is calculated following the procedure in Love, Huber, and Anders, 2014. For example, considering the following binned count data for three genes across three bins,

	Bin 1	Bin 2	Bin 3
Gene 1	100	150	200
Gene 2	200	300	250
Gene 3	300	450	500

To adjust for library size differences, we first calculate the geometric mean for each gene, followed by the size factors for each bin.

Geometric Mean: The geometric mean of counts for each gene, y_i, is given by:

$$\text{GeoMean}_{y_i} = \exp\left(\frac{1}{N}\sum_{j=1}^{N}\log(\text{Count}_{y_i,j})\right) \tag{1}$$

For Gene 1 (y_1), the calculation is:

$$\text{GeoMean}_{y_1} = \exp\left(\frac{\log(100)+\log(150)+\log(200)}{3}\right) \tag{2}$$

Size Factor: The size factor for each bin is the median of the ratios of its counts to the geometric means for each gene:

$$\text{SizeFactor}_{\text{Bin}} = \text{median}\left(\frac{\text{Count}_{\text{Gene,Bin}}}{\text{GeoMean}_{\text{Gene}}}, \ldots\right) \tag{3}$$

scMaSigPro uses the '`estimateSizeFactorsForMatrix()`' from DESeq2 (Love, Huber, and Anders, 2014) to compute this offset, resulting in the following poly-GLM model:

$$\log(\text{Expected Count}_{\text{Gene}}) = \beta_0 + \beta_1 X + \log(\text{SizeFactor}) \tag{4}$$

where log(SizeFactor) is the offset. A positive offset adjusts for a higher baseline level of counts, while a negative offset adjusts for a lower baseline level. Hence, *scMaSigPro* can directly process raw counts by setting the 'offset' parameter in the '`sc.p.vector()`' function to `TRUE`. This allows for the direct incorporation of library size normalization in the analysis. Users can also employ custom normalization strategies by adjusting the model distribution settings within '`sc.p.vector()`'.

2 Benchmarks

We used *Splatter*, which employs a Gamma-Poisson distribution to simulate scRNA-Seq data (Zappia, Phipson, and Oshlack, 2017). Splatter simulates a series of steps between the start and end of a path and randomly assigns each cell to a **Step**. As *Pseudotime* values are arbitrary and only reflect the order of the cells, we can easily extrapolate *Step* (hereinafter, '*Pseudotime*') to be treated as *Pseudotime* for the evaluation. Additionally, just like *Pseudotime*, the lower values for *Step* denote immature cells with a value close to 0, followed by the cells in the transitional stage *Pseudotime* > 0, and finally, the mature cells. Additionally, Splatter simulates **Groups**, which corresponds to the branching paths in which each cell exists ('simulated branching Paths').

2.1 Bifurcating Paths and Base Simulation

We rationalize that complex topological structures, such as trifurcations or multifurcations, can typically be simplified into a more fundamental bifurcation structure, with the notable exception of cyclic topologies. A bifurcation topology is characterized by two distinct branching paths and a critical point along the *Pseudotime* axis, where genes exhibit differential dynamic expression, and diverging lineages emerge (Supplementary Figure 3). Any branches can be treated as a reference, and the expression patterns can be compared against the other branches. This unlocks the analysis with *maSigPro* of expression patterns that change along *Pseudotime* or both branching paths and *Pseudotime* simultaneously.

As a base simulation, we simulated 2000 features (genes) and 3000 cells distributed across two branching paths. This resulted in a dataset with approximately 1500 cells per branching path, uniformly spanning from 0 to 1500, with approximately one cell per **Step/Pseudotime**. The number of cells compared to the number of genes was deliberately kept high to resemble the typical scRNA-Seq experiment.

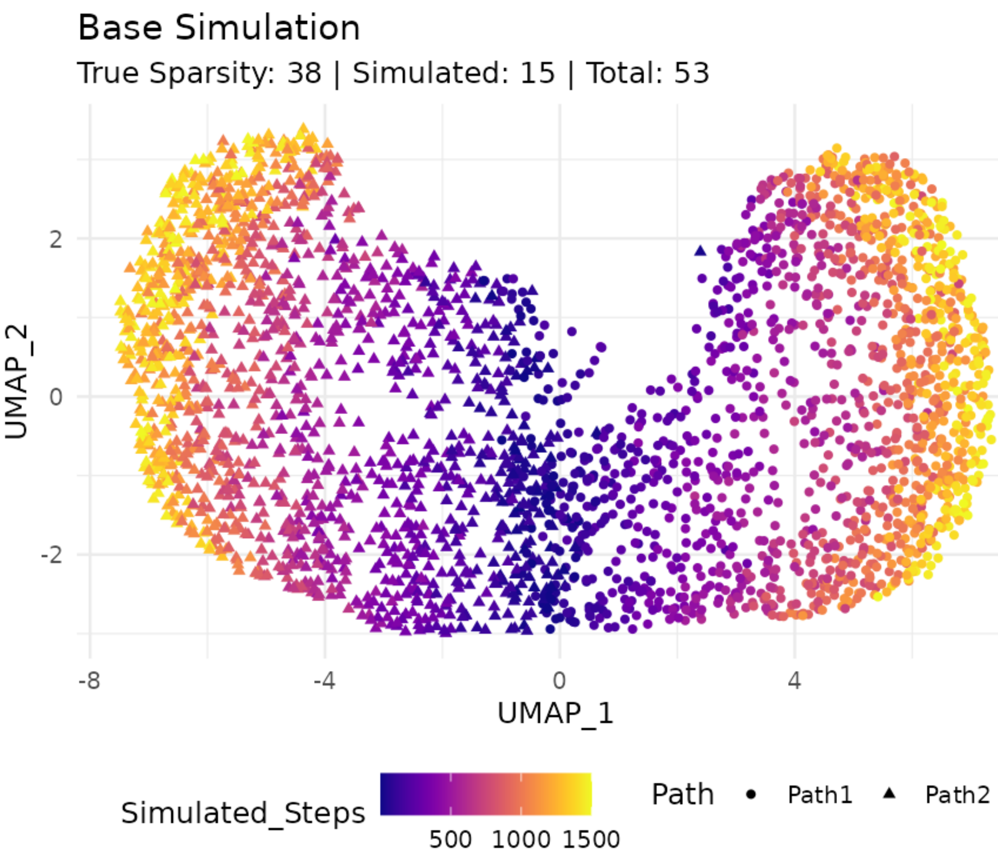

Supplementary Figure 3: Representation of the base simulated bifurcating trajectory using Splatter using UMAP. Each cell is coloured by the associated value of the simulated *Pseudotime*, and different shapes depict different simulated branching paths.

2.2 Varying levels of Zero-Inflation/Sparsity

High Zero-inflation (ZI) (or sparsity) is a major characteristic of scRNA-Seq data, which is a consequence of capture-induced noise (technical drop-outs) and the inherent biology (biological drop-outs) (Jiang et al., 2022). We simulated synthetic datasets with increasing sparsity levels, i.e., from 60% to 90% in intervals of 10. Specifically, we adjusted the '`dropout.shape`' parameter in the '`splatSimulate()`' function to add artificial zero-inflation (technical dropouts). The true zero-inflation of the dataset (biological dropouts) was learned from the real dataset (section 4) mentioned before and set to a constant of 38%. The sparsity level for each dataset combined the simulated sparsity and true sparsity, resulting in four datasets (Supplementary Figure 4A to D).

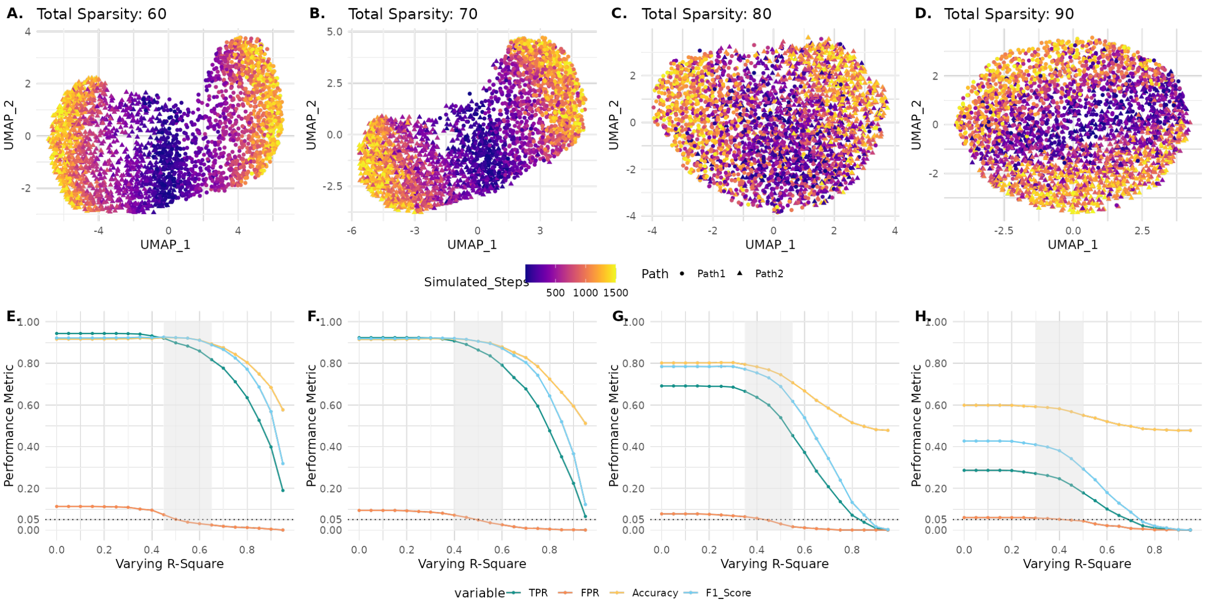

Supplementary Figure 4: (A to D) Representation of the increasing ZI levels in simulated bifurcating trajectories using the UMAP. Each cell is coloured by the associated value of the simulated *Pseudotime*, and different shapes depict different simulated branching paths. (E to H) *scMaSigPro* performance in each simulated dataset as a function of increasing R^2 values, obtained with the ROCR package Sing et al., 2005

2.3 Varying capture bias of cells (Skewness)

To assess the impact of Skewness in cell density distributions along the trajectory path, we simulated four datasets. In two of these datasets, cells were simulated to be more densely distributed at the beginning of the paths (Skew 0.9 and 1), while in the other two cells were simulated to concentrate at the end of the trajectory (Skew 0 and 0.1) (5-A to D). For such datasets, we adjusted the `path.skew` parameter in the `splatSimulate()` and set the ZI level of 60%.

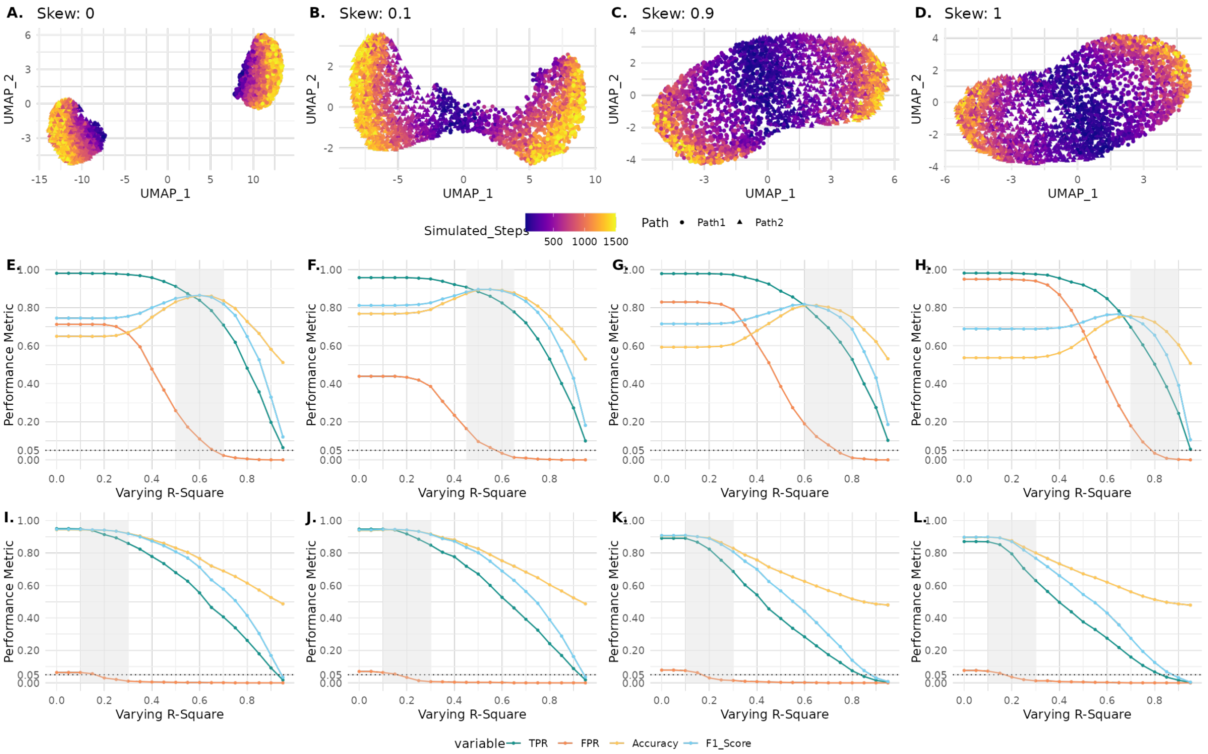

Supplementary Figure 5: (A to D) Representation of the extreme skewness in simulated bifurcating trajectories using the UMAP. Each cell is coloured by the associated value of the simulated *Pseudotime*, and different shapes depict different simulated branching paths. (E to H) *scMaSigPro* performance in each simulated dataset as a function of increasing R^2 values, obtained with the ROCR package (Sing et al., 2005). (I to L) *scMaSigPro* performance in the previous datasets obtained with the *scMaSigPro* `split_bins` function enabled.

2.4 Unequal Length of Branching Paths

In our final set of simulations, we generated four datasets to represent variable developmental times. To model such scenarios, we adjusted the `group.prob` for the number of cells within a branching path and the `path.nSteps` for adjusting the number of steps a cell takes to reach its end cell state (Range of simulated *Pseudotime*) (Supplementary Figure 6A to D). The ZI level is 60% for all the datasets.

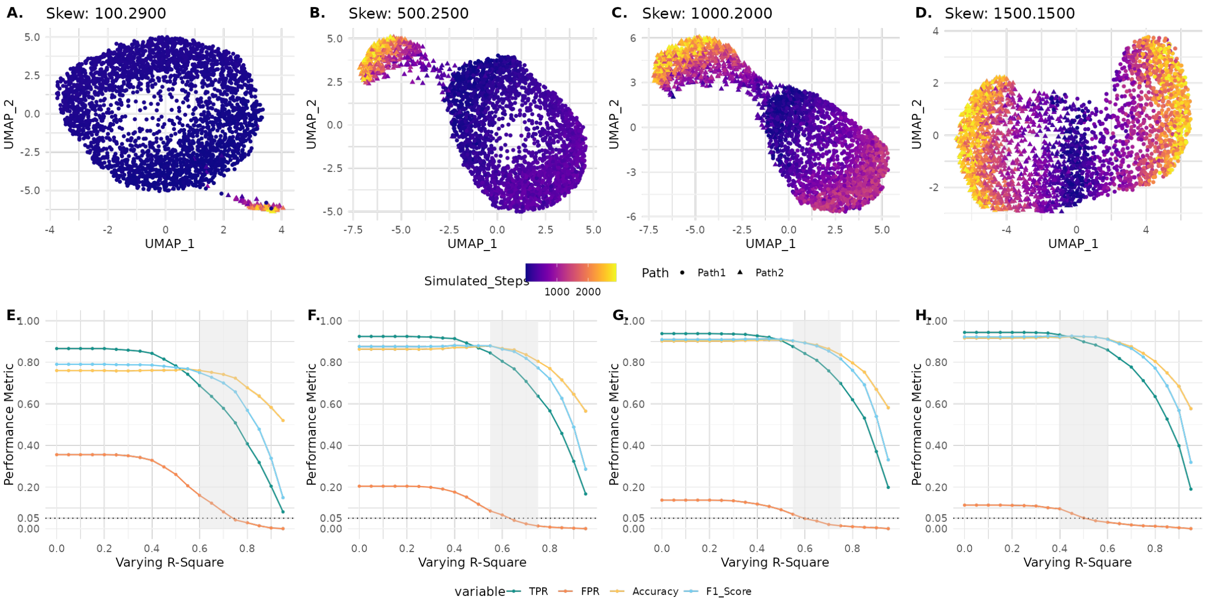

Supplementary Figure 6: (A to D) Representation of the different lengths of the branching paths in simulated bifurcating trajectories using the UMAP. Each cell is coloured by the associated value of the simulated *Pseudotime*. Different shapes depict different simulated branching paths. (E to H) *scMaSigPro* performance in each simulated dataset as a function of increasing R^2 values obtained with the ROCR package Sing et al., 2005.

3 Comparison with tradeSeq

3.1 Simulation of Data

To compare and evaluate the difference between the tradeSeq and *scMaSigPro*, we simulated a dataset with 3000 cells and 2000 genes with parameters learned from the real dataset as stated above. Additionally, parameters were adjusted to produce datasets with different path lengths and skewness, keeping ZI at 60%. Globally, 30% of the genes were simulated to be DE. `path.nonlinearProb` parameter in Splatter forces the simulated DE genes to follow a non-linear expression pattern along the simulated *Pseudotime*. 9% of the total genes (i.e. 30% of the DE genes) were simulated to follow non-linear expression patterns between a path's start and end points (Supplementary Figure 7).

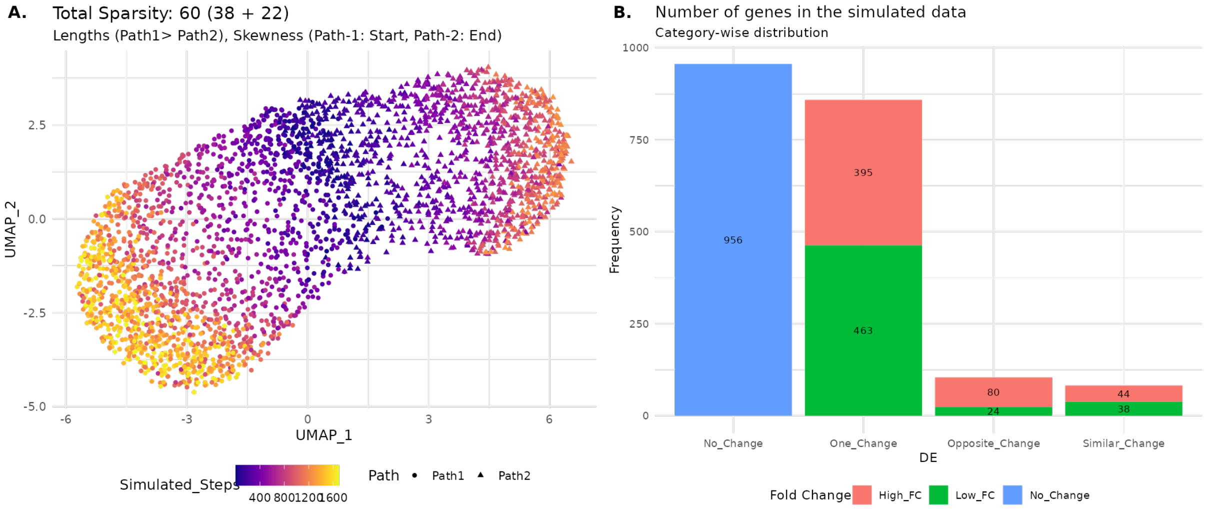

Supplementary Figure 7: (A) UMAP of the simulated dataset used for the comparative evaluation of tradeSeq and *scMaSigPro*. (B-E) Bar plot of the genes, representing their expression patterns category.

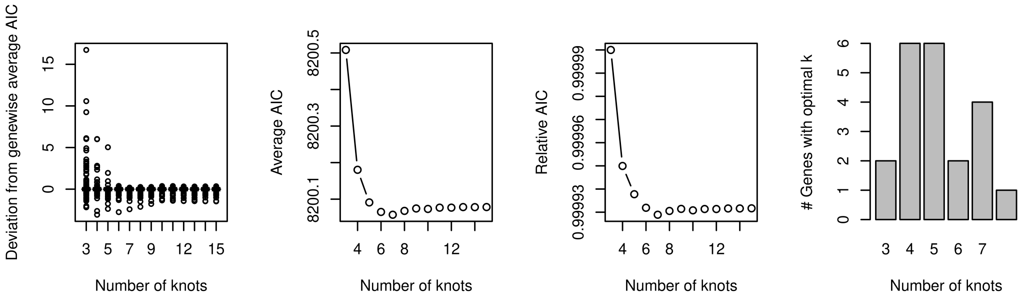

Supplementary Figure 8: Result of evaluteK() from tradeSeq. The optimal number of K is chosen to be five as the relative change in AIC is highest from 4 to 5.

3.2 Evaluation with iCobra

To compare the gene lists obtained by running tradeSeq (`patternTest()` and `diffEndTest()`) and *scMaSigPro*, we used iCOBRA framework (interactive comparative evaluation of binary classification and ranking methods) Soneson and Robinson, 2016. The iCOBRA framework offers methods to evaluate gene lists from binary classification methods against a ground truth.

3.3 False Negatives

We explored the features that were not detected by any of the methods but were simulated to have a change in expression along *Pseudotime* i.e. False Negatives (Supplementary Figure 9 and 10).

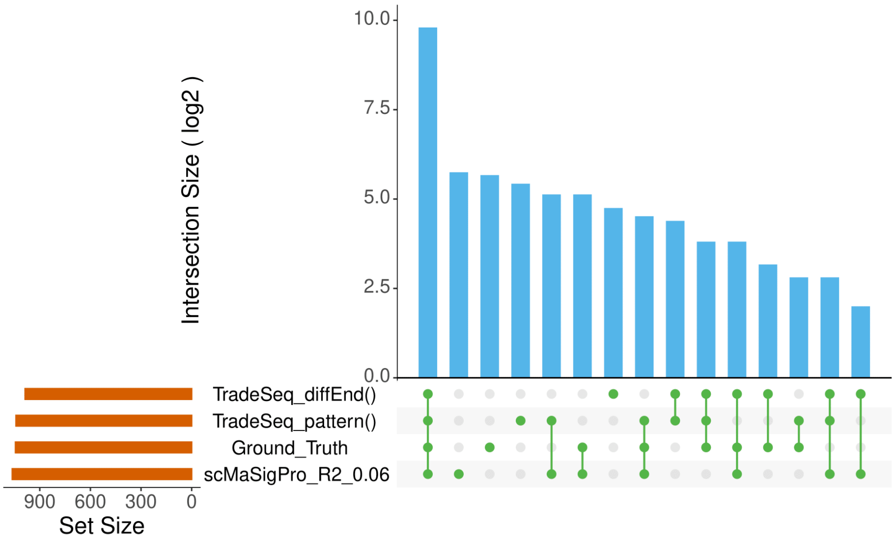

Supplementary Figure 9: UpSet Plot showing the intersection of the DE predictions made by *scMaSigPro* and *tradeSeq* and the ground truth.

We observed that *scMaSigPro* failed to identify genes with low fold changes 10-B, specifically when the gene is DE in only one of the branching paths. Such patterns are picked up by the tradeSeq's `diffEnd()` and `pattern()`. On the contrary, tradeSeq failed to identify DE genes with low and high fold change and different patterns, representing a more erratic behaviour. Interestingly, both methods successfully identified genes with opposite trends in both the lineages.

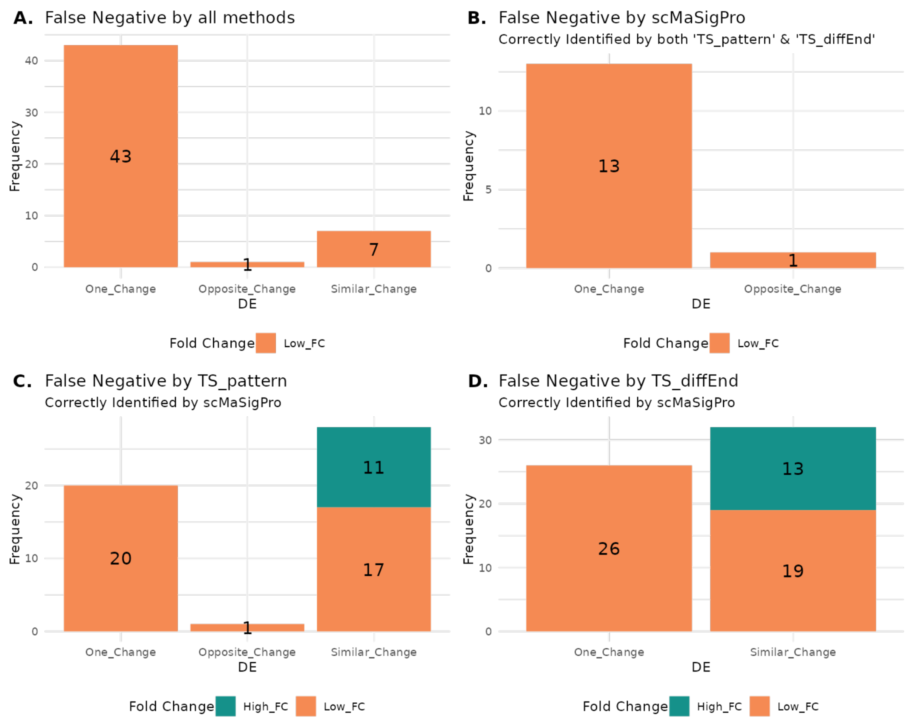

Supplementary Figure 10: Bar plots showing the intersection of the False Negatives by tradeSeq and *scMaSigPro*.

3.4 Evaluation of computational running time and scalability

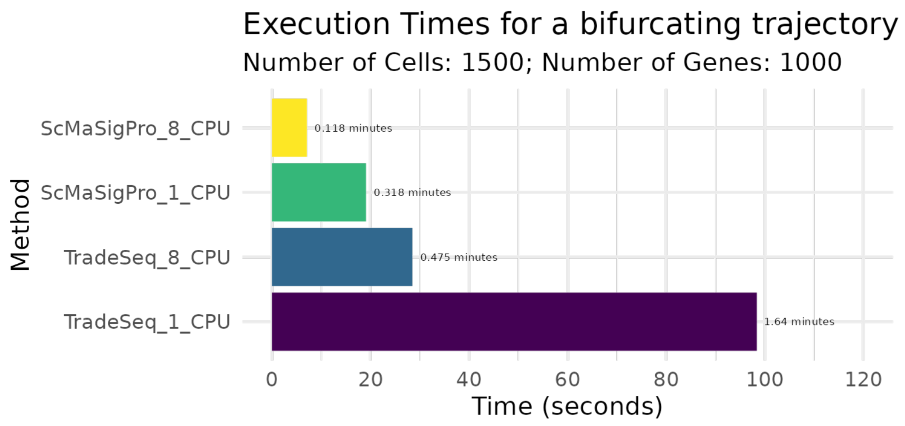

Supplementary Figure 11: Bar plot showing computing time for *scMaSigPro* and *tradeSeq* with 8 CPU cores.

3.4.1 Environmental Impact of Algorithm

Table 1 shows the impact of running both tradeSeq and *scMaSigPro* on a personal computer with 32 Gb memory based in Spain (Lannelongue, Grealey, and Inouye, 2021, calculator.green-algorithms.org/) on the 1500 cells dataset. *scMaSigPro* not only alleviates the computational bottlenecks often encountered in analysing large-scale data but also significantly reduces the carbon footprint of the analysis (Lannelongue, Grealey, and Inouye, 2021), as tradeSeq produces five times the carbon footprint when run with a single core.

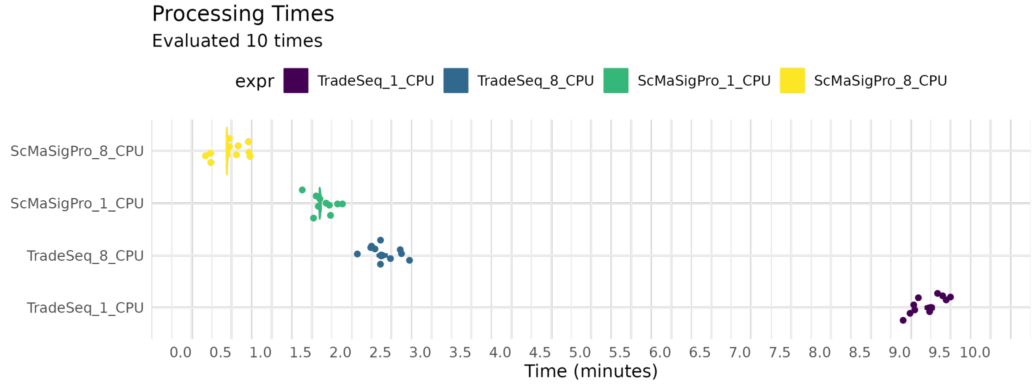

Supplementary Figure 12: Plots showing the runtime in minutes for each algorithm on a larger dataset of 6000 cells. Each method was run 10 times using the R-microbenchmark framework. Each point in the plot represents an individual run.

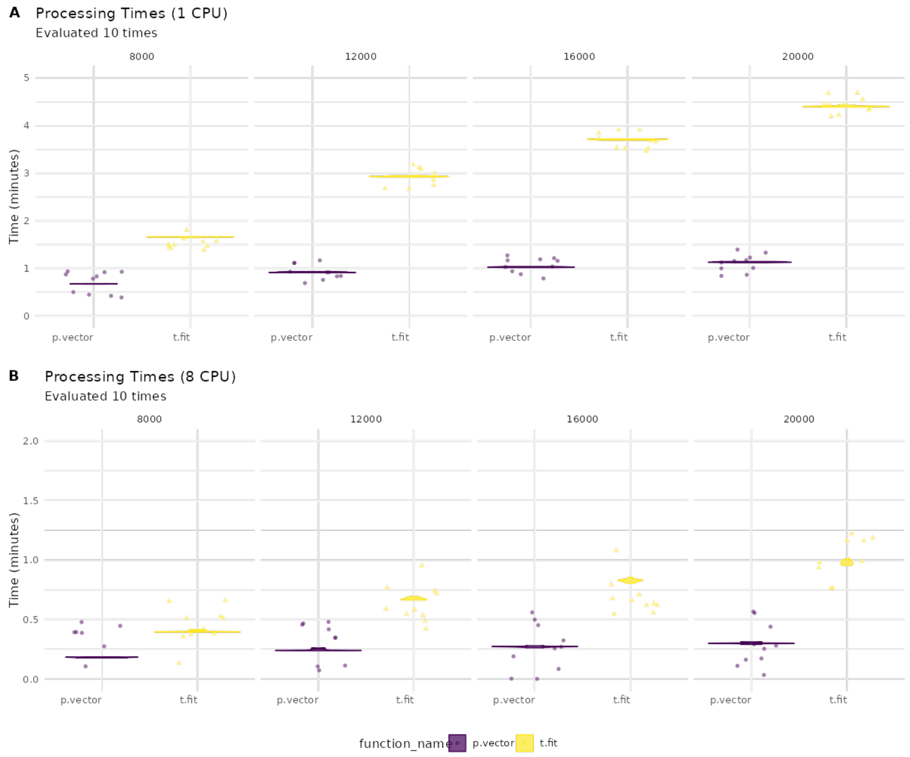

Supplementary Figure 13: Plots showing the runtime in minutes for each 'sc.pvector()' and 'sc.t.fit()' function with an increasing number of cells. Each function was evaluated 10 times with increasing total cells using the R-microbenchmark framework. Each branching path in the dataset contained half the number of total cells. Each point in the plot represents an individual run.

Table 1: Environmental impact of algorithm, calculated using calculator.green-algorithms.org/ (Lannelongue, Grealey, and Inouye, 2021)

Algorithm	CPU	Time (Minutes)	Carbon Footprint (mg CO2e)	Energy (Wh)	Carbon sequestration (Tree Months)
scMaSigPro	Single	0.3	91.60	5.36e-01	9.99e-05
scMaSigPro	8	0.1	30.53	1.79e-01	3.33e-05
tradeSeq	Single	1.7	519.09	3.04	5.66e-04
tradeSeq	8	0.4	122.14	7.14e-04	1.33e-04

4 Analysis of Public Data

4.1 Data Preparation with Seurat

We discarded cells that did not have a minimum threshold of 10 detected genes (`min.cells = 10`), a minimum gene count of 1000 (`min.features = 1000`) and more than 10% reads mapping to mitochondrial gene set (table 3). As per methods in Setty et al., 2019, we also removed the cell cycle effects from each of the donors, using the genes enriched with 'DNA-Replication (S-Phase)(GO:0006260)', 'Mitotic M phase (G2M-Phase)(GO:0000087)', 'M-Phase (G2M-Phase)(GO:0000279)', 'Chromosome Segregation (G2M-Phase) (GO:0007059)', 'Organelle Fission (G2M-Phase) (GO:0048285)'.

Table 2: Summary of Cell Types evaluated

Cell Types, ontologies and markers from Azimuth Human Bone Marrow Reference		
Cell Type	Short Name	Marker Gene
Early Erythroid	Early E	CNRIP1, GATA2, ITGA2B, TFR2, GATA1, KLF1, CYTL1, MAP7, FSCN1, APOC1
Erythroid Megakaryocyte Progenitor	EMP	MYCT1, CRHBP, NPR3, AVP, GATA2, HPGDS, CYTL1, CRYGD, IGSF10, PBX1
Granulocyte Monocyte Progenitor	GMP	SERPINB10, RNASE3, MS4A3, PRTN3, ELANE, AZU1, CTSG, RNASE2, RETN, NPW
Hematopoeitic Stem cell	HSC	CRHBP, AVP, MYCT1, BEX1, NPR3, CRYGD, MSRB3, CD34, NPDC1, MLLT3
Lymphoid Primed Multipotent Progenitor	LMPP	AVP, CRHBP, C1QTNF4, BEX1, NPR3, CD34, NPW, SMIM24, CSF3R, NPDC1
Progenitor Megakaryocyte	Prog Mk	CLEC1B, SPX, WFDC1, ANXA3, CMTM5, SELP, RBPMS2, ARHGAP6, GP9, LTBP1
Common Lymphoid Progenitor	CLP	ACY3, PRSS2, C1QTNF4, SPINK2, SMIM24, NREP, CD34, DNTT, FLT3, SPNS3
Precursor Plasmacytoid Dendritic Cell	pre-pDC	SCT, SHD, LILRA4, LILRB4, PTPRS, TNNI2, PLD4, SPIB, IRF8, TNFRSF21
Precursor Myeloid Dendritic Cell	pre-mDC	ENHO, CLEC10A, RNASE2, PLBD1, FCER1A, IGSF6, MNDA, SAMHD1, ALDH2, PAK1
Progenitor B	pro B	CYGB, UMODL1, EBF1, MME, VPREB1, DNTT, IGLL1, UHRF1, BLNK, AGPS

4.2 Cell Type Annotation & Sub-Sampling

The gene markers used for the cell-type identification and analysed cell types are listed in table 3. After the cell-type annotation with Azimuth, we removed the cells labelled as T cells, CD4 memory cells and fully differentiated cells (i.e. 'BaEoMa', 'Stromal', 'transitional B') following the methods of the Setty et al., 2019 (Supplementary Figure 14). Lastly, the top 6000 highly variable genes were selected using Seurat's '`FindVariableFeatures()`' and used for the TI analysis.

4.3 Trajectory Inference

Trajectory Inference was performed using Monocle3 (Cao et al., 2019). Each dataset, corresponding to different donors, was analyzed independently. The process began by creating Monocle3 CDS objects for each of the three donors and transferring the UMAP embeddings from the Seurat objects to these Monocle3 objects. For the selection of the root cell, we chose the principal point detected by Monocle3, characterized by the highest population of 'HSCs' & 'LMPP' (only Donor-2) in UMAP space. Finally, *Pseudotime* computations were carried out for all three donors (Supplementary Figure 15-A to F). We evaluated different lineages for different donors as tabulated in table 3.

4.4 scMaSigPro Analysis

4.4.1 Setting Polynomial GLM

Initially, 'Cell Data Set' (CDS) S4 objects from Monocle3 were converted to 'scMaSigPro Class' Objects (scmpObject). *scMaSigPro* offers a helper function '`as_scmp()`' to directly convert and subset widely used S4 objects

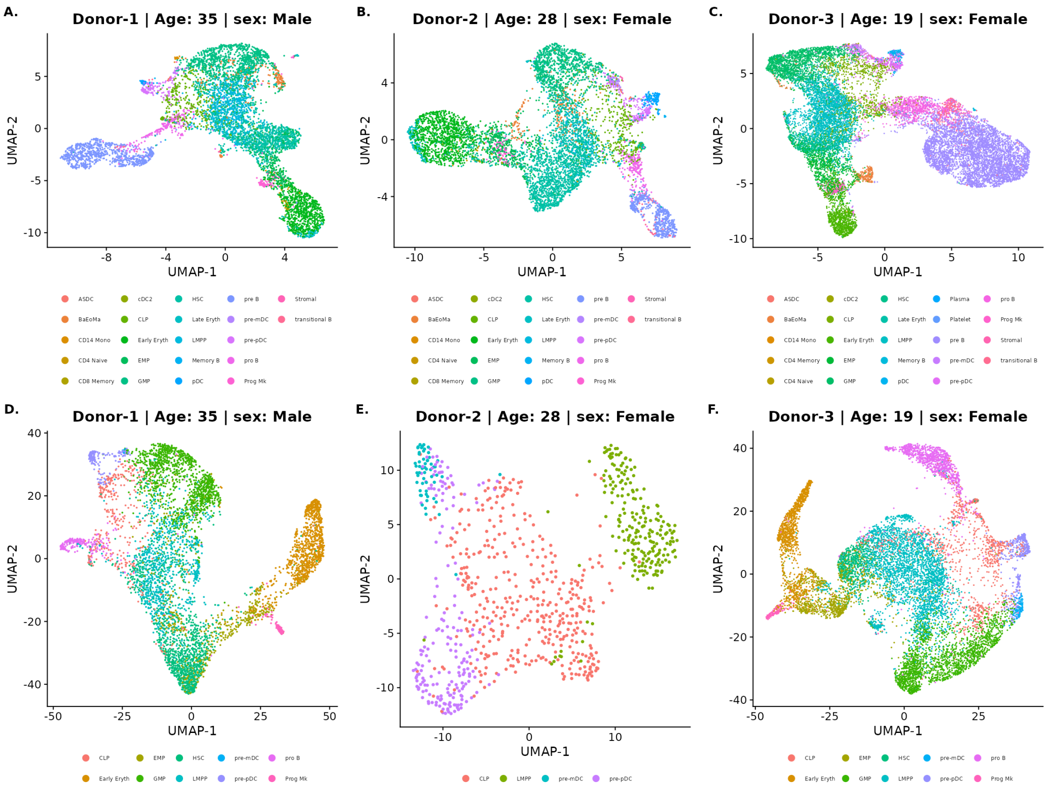

Supplementary Figure 14: (A-C) Representation of the annotated cell type for each donor by Azimuth on UMAP calculated by Seurat. (D-F) Sub-sampled data used for TI with Monocle3

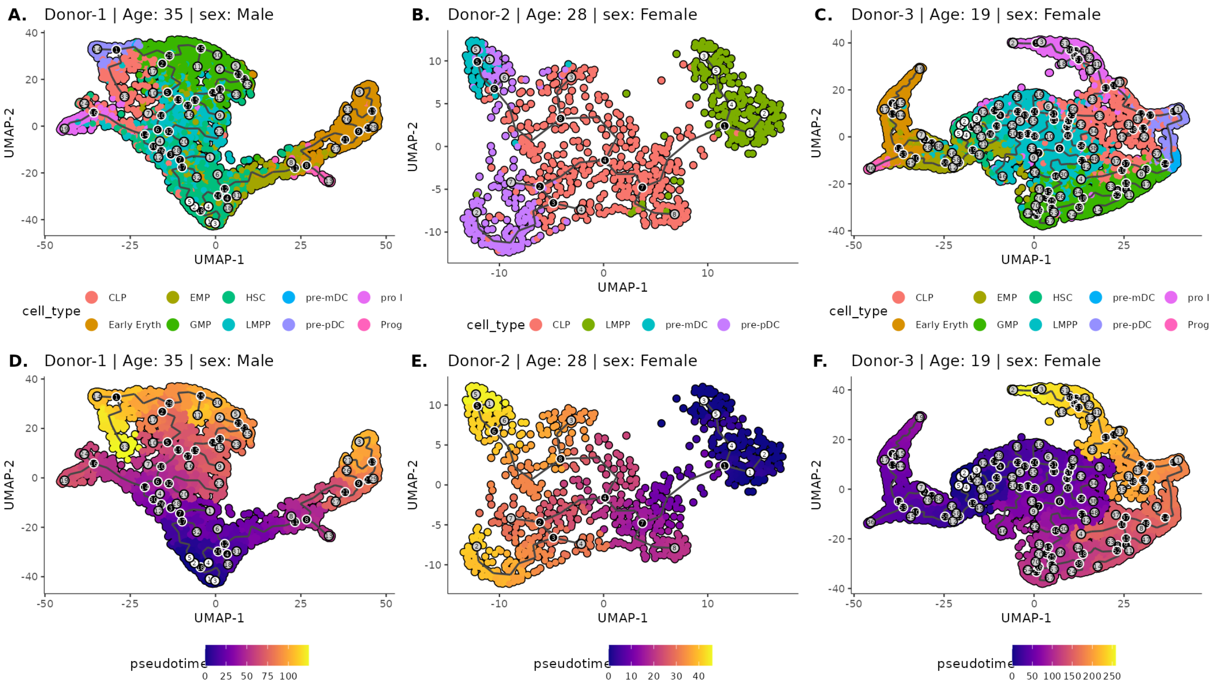

Supplementary Figure 15: Representation of the trajectory inferred using Monocle3. A-C trajectory on the UMAP coloured by cell types. D-F trajectory on the UMAP coloured by *Pesudotime*. Principal points are highlighted in all the UMAPs.

Table 3: Summary of filtering and selection performed on the raw counts of the Setty et al., 2019 dataset.

Filtering of raw counts to generate processed counts			
Donor	Donor-1	Donor-2	Donor-3
Raw Feature Barcode Matrix from Cell Ranger	No of barcodes: 6,998<br>No of Features: 62,703	No of barcodes: 7,812<br>No of Features: 62,703	No of barcodes: 17,898<br>No of Features: 62,703
Filtering Cells with Seurat	No of barcodes: 6,602<br>No of Features: 25,961	No of barcodes: 6,982<br>No of Features: 26,844	No of barcodes: 16,272<br>No of Features: 26,963
Selection of Cell Types per donor			
Sub-Sampled Cell Types	GMP, Erythrocyte, HSC, LMPP, EMP, Prog Mk, CLP, pre-pDC pre-mDC, pro B	LMPP, CLP, pre-pDC, pre-mDC	GMP, Erythrocyte, HSC, LMPP, EMP, Prog Mk, CLP, pre-pDC pre-mDC, pro B
No of HVG selected with Seurat	6,000	6,000	6,000
No of Cells used in TI with Monocle3	5,328	880	10,242
Root Cell Type	HSC	LMPP	HSC
Selection of branching paths per donor (After Cell Type Inference and TI)			
Total Number of Cells	195	360	3,627
Branch-1	EMP to Early Erythrocyte	CLP to pre-mDC	HSC to EMP
Branch-2	EMP to Prog Mk	CLP to pre-pDC	HSC to GMP

in single-cell data analysis directly to scmpObject. In the case of CDS objects, *scMaSigPro* also has a built-in shiny selection wizard, which the user can use to interactively subset the trajectory for branching paths and then use it in the *scMaSigPro* pipeline.

After selecting the branching paths and creating the scmpObject, the '`sc.squeeze()`' function was applied to perform the binning. Specifically, we used the 'Sturges' method with a `drop_factor` of 1 for all the donors (Supplementary Figure 16). We set a polynomial order three to set up cubic GLMS for all the donors (eq 5, 6, 7). For Setty et al., 2019 dataset, we changed the distribution to *Gaussian* because the Seurat scaled the counts during the cell-cycle effect correction procedure.

Poly-GLM for Donor-1

$$\begin{aligned}\beta_0 + \beta_1 \times \text{EMP\_ProgMkvsEMP\_EarlyErythrocyte} + \beta_2 \times b\text{Pseudotime} + \beta_3 \times b\text{Pseudotime} \times \text{EMP\_ProgMk}+\\ \beta_4 \times b\text{Pseudotime}^2 + \beta_5 \times b\text{Pseudotime}^2 \times \text{EMP\_ProgMk} + \beta_6\\ \times b\text{Pseudotime}^3 + \beta_7 \times b\text{Pseudotime}^3 \times \text{EMP\_ProgMk}\end{aligned} \tag{5}$$

Poly-GLM for Donor-2

$$\begin{aligned}\beta_0 + \beta_1 \times \text{CLP\_pre\_pDCvsCLP\_pre\_mDC} + \beta_2 \times b\text{Pseudotime} + \beta_3 \times b\text{Pseudotime} \times \text{CLP\_pre\_pDC}+\\ \beta_4 \times b\text{Pseudotime}^2 + \beta_5 \times b\text{Pseudotime}^2 \times \text{CLP\_pre\_pDC} + \beta_6\\ \times b\text{Pseudotime}^3 + \beta_7 \times b\text{Pseudotime}^3 \times \text{CLP\_pre\_pDC}\end{aligned} \tag{6}$$

Poly-GLM for Donor-3

$$\begin{aligned}\beta_0 + \beta_1 \cdot \text{HSC\_GMPvsHSC\_EMP} + \beta_2 \cdot b\text{Pseudotime} + \beta_3 \cdot b\text{PseudotimexHSC\_GMP}\\ +\beta_4 \cdot b\text{Pseudotime}^2 + \beta_5 \cdot b\text{Pseudotime}^2 \times \text{HSC\_GMP} + \beta_6 \cdot b\text{Pseudotime}^3+\\ \beta_7 \cdot b\text{Pseudotime}^3 \times \text{HSC\_GMP}\end{aligned} \tag{7}$$

4.4.2 Model Fitting and Selection of Genes

After running the '`sc.p.vector()`' function, genes were selected based on the adjusted p-values (with the Benjamini-Hochberg procedure) with a significance level of 0.05. Further, the significant terms were selected

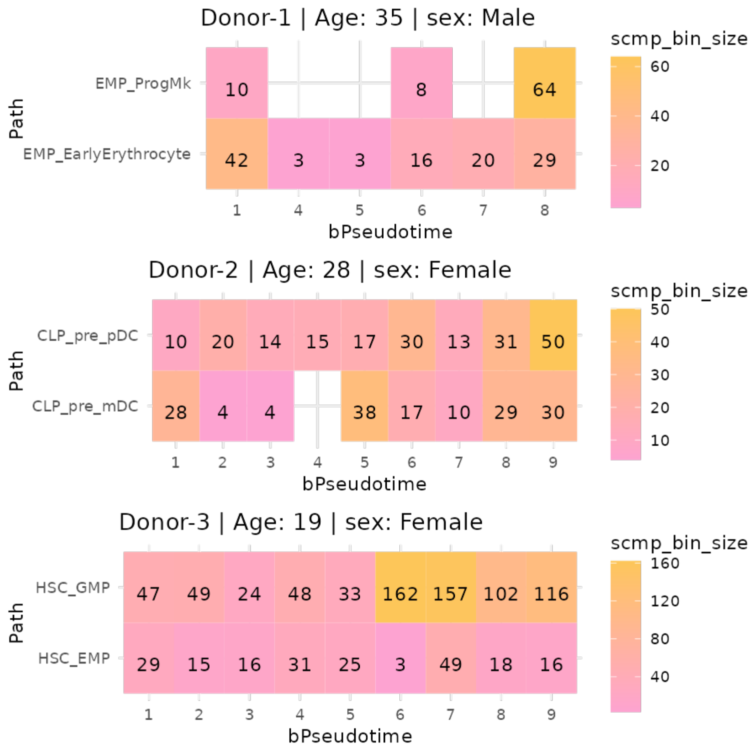

Supplementary Figure 16: Visualization of the number and size of the bins after the 'sc.squeeze()'.

from the polynomial models with '`sc.t.fit()`'. A summary of the genes with non-flat profiles is given in table 4. Significant DE genes were called, passing a R^2 threshold of 0.7 for each of the donors (table 4). The Significant DE genes from each of the donors were manually inspected for the presence of known gene markers listed in table 2. Then, we examined the trend of 4 significant markers using the 'plotTrend()' function for each of the donors (Supplementary Figure 19).

Table 4: Number of Genes with Non-Flat Profiles and Significant Genes

Donor	Number of Genes with non-flat profiles	Number of Genes Passing the $R^2 \geq 0.7$
Donor-1	300	300
Donor-2	526	512
Donor-3	2,259	1,476

4.4.3 Clustering Gene Expression Trends

'clusterProfiler' was used to perform an overrepresentation analysis of Gene Ontology Biological Process terms in the list of *scMaSigPro* detected genes. All experimentally captured genes were used as the background. The enrichment significance level was set to 0.05 (Supplementary Figure 18). The enrichment results were visualized with 'enrichplot' using `dotplot()`.

As expected, genes involved in cell fate decisions were related to the development of cell lineages. For Donor-1, terms like 'response to wound healing', 'blood coagulation', and 'platelet activation' were enriched, which reveals the differentiation process of the HSPCs to Megakaryocyte Lineage, resulting in platelets (Supplementary Figure 18-A). For Donor-2, enriched terms included 'leukocyte activation', 'myeloid-leukocyte activation' and terms related to immune responses, representing the development of myeloid-derived Dendric cells (mDC) as they play a part in the immune response regulation (Figure 18-B). Finally, For Donor-3, for which we evaluated the gene list from the erythroid lineage, terms like 'myeloid cell homeostasis', 'erythrocyte differentiation' and 'erythrocyte homeostasis' were enriched (Supplementary Figure 18-C).

After obtaining the gene list with significant expression changes among branching paths and *Pseudotime*, genes were clustered according to their expression trends in each of the branching paths (Supplementary Figure 17). This was performed with the 'sc.cluster.trend()', which calls the '`see.genes()`' and '`PlotProfile()`' from *maSigPro*. The clustering option was set to 'hclust', and the expected number of clusters was set to 6.

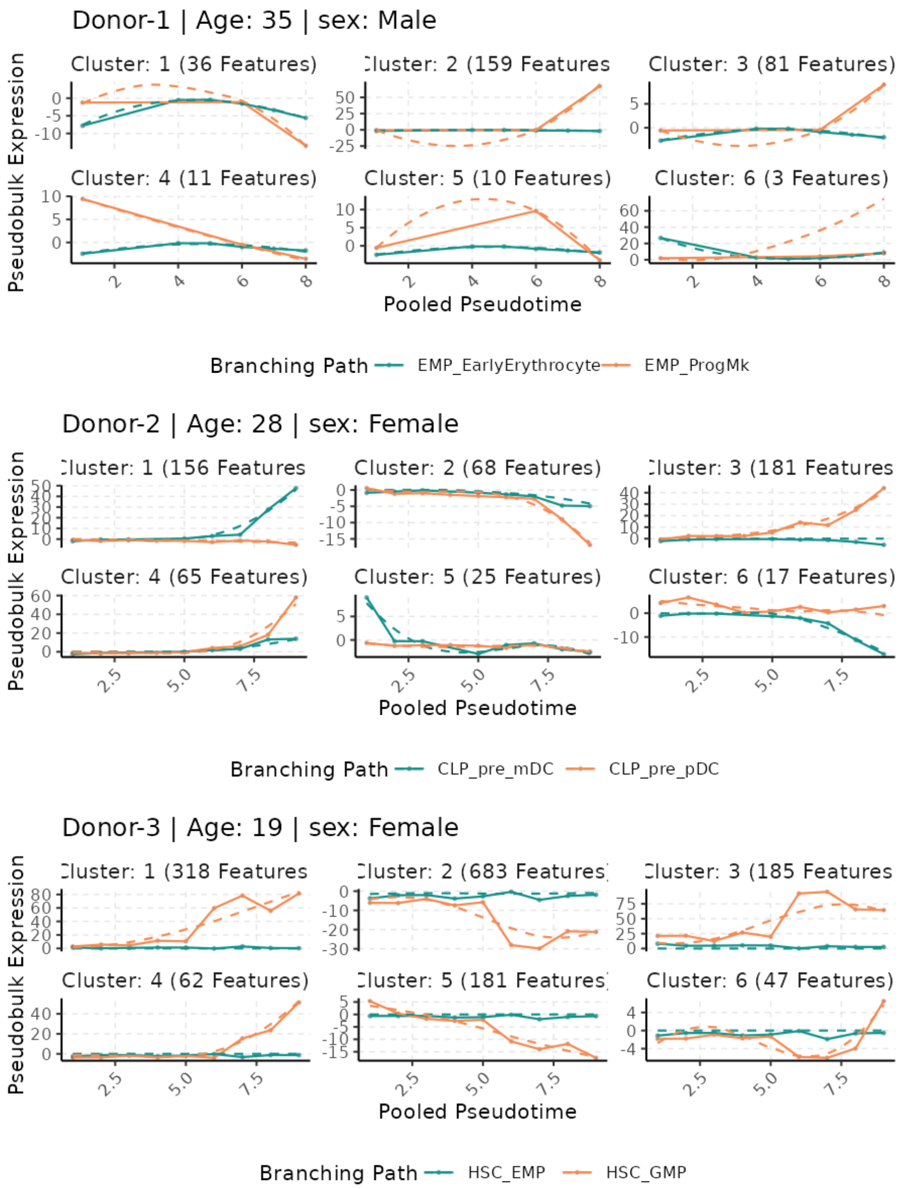

Supplementary Figure 17: Clustering of the Gene Expression Trends. For Donor-1, we selected clusters 2 and 3 (240 genes), showing an upward trend in Megakaryocyte Lineage. For Donor-2, we selected cluster 1 (156 genes), showing an upward trend in the Myeloid-Dendritic Lineage, and for Donor-3, we selected clusters 1, 3 and 4 (565 genes) with an upward trend in GMPs.

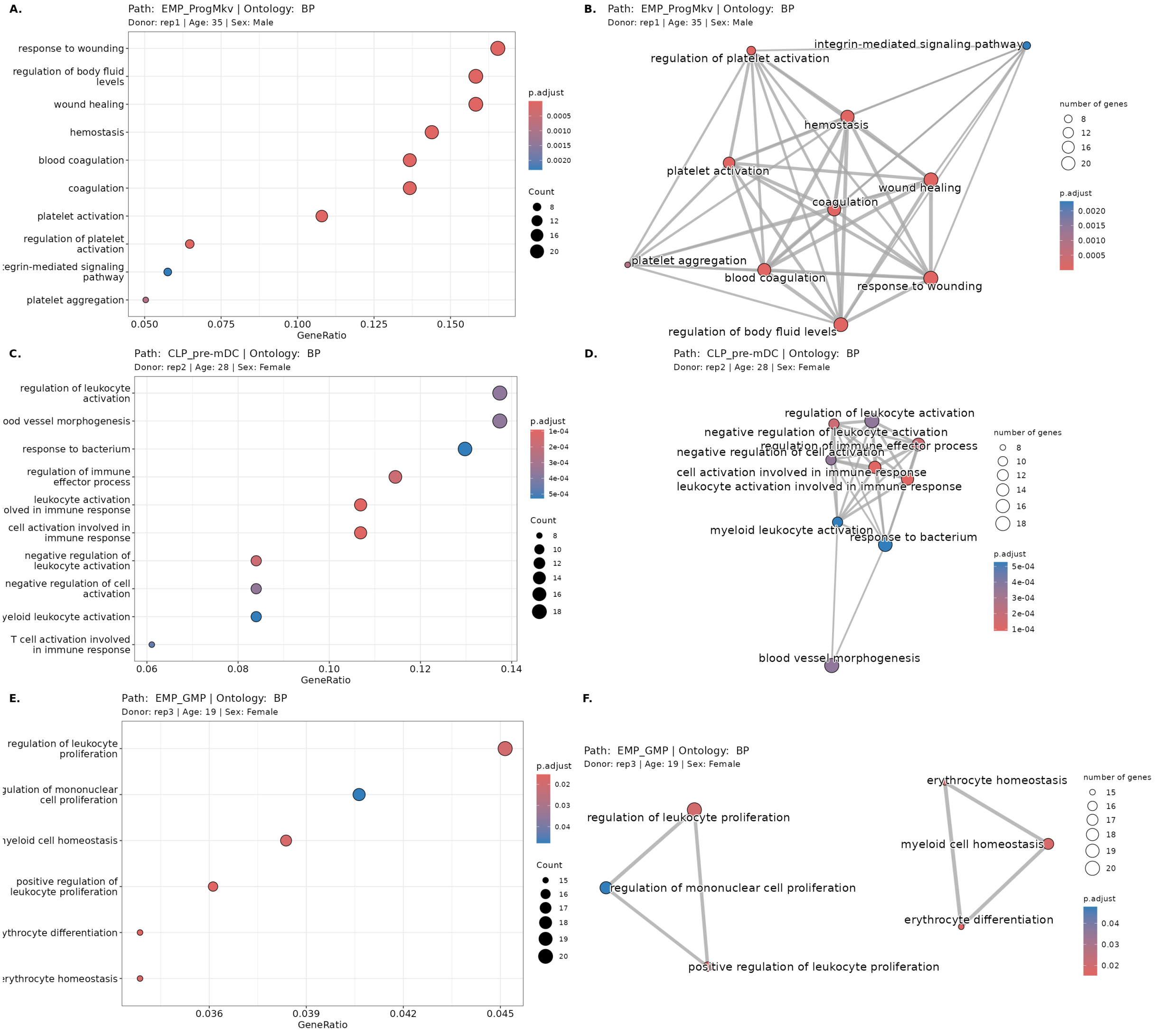

Supplementary Figure 18: Dotplots for topmost enriched GO terms for each of the within the Setty et al., 2019 dataset.

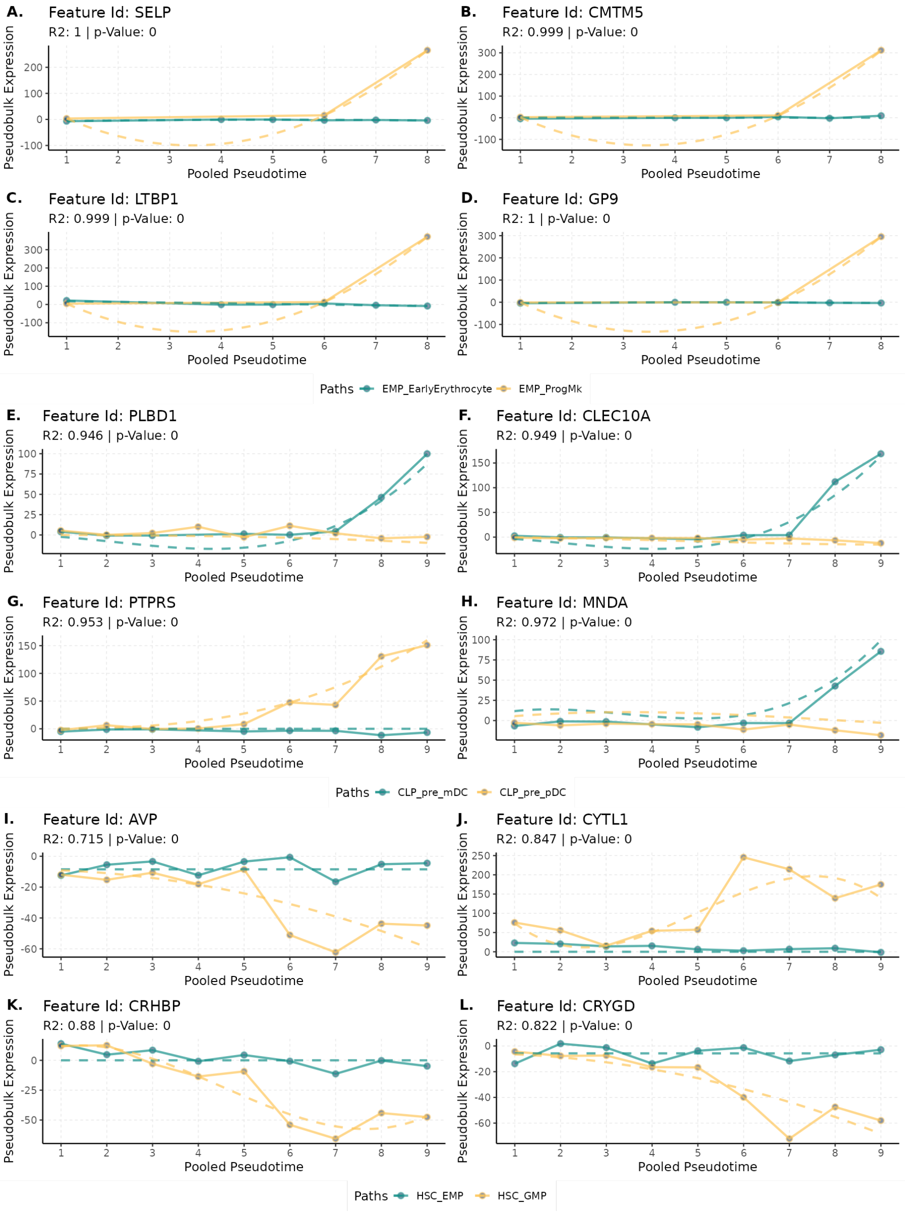

Supplementary Figure 19: Trends of the known genes markers identified as significant and plotted using *scMaSigPro*. (A-D) Donor-1, (E-H) Donor-2, (I-L) Donor-3, from Setty et al., 2019. The dashed line is the polynomial GLM of the *scMaSigPro*, and the solid lines connect the actual values of the expression along the *Pseudotime*.

5 Additional Information

1. **scMaSigPro**('https://github.com/BioBam/scMaSigPro'): R-Package, to be submitted to CRAN.
2. **Additional Table-1**('Additional_Table_1_All_Performance_Measures_Results.xlsx'): Contains performance metric for each of the benchmarks.
3. **Additional Table-2**('Additional_Table_2_Mechanistic_Analysis_Results.xlsx'): Contains a list of significant genes for each donor found by *scMaSigPro*. It also contains the list of annotated clusters and the results of GO enrichment analysis for each of the donors from Setty et al., 2019.
4. **Docker Container**('https://hub.docker.com/r/spriyansh29/sc_masigpro'): Docker container to reproduce the results.
5. **R-Scripts**('https://github.com/biobam/scMaSigPro_Supp/'): Contains all the R-scripts to reproduce the results.
6. **Simulated Data**('https://zenodo.org/records/12568922'): All the data is submitted to Zenodo for review and will be publicly available.

References

Cao, Junyue et al. (Feb. 28, 2019). "The single-cell transcriptional landscape of mammalian organogenesis". In: *Nature* 566.7745, pp. 496–502. ISSN: 0028-0836, 1476-4687.

Jean Hausser and Korbinian Strimmer (Oct. 2, 2021). *entropy: Estimation of Entropy, Mutual Information and Related Quantities.* Version 1.3.1.

Jiang, Ruochen et al. (Jan. 21, 2022). "Statistics or biology: the zero-inflation controversy about scRNA-seq data". In: *Genome Biology* 23.1, p. 31. ISSN: 1474-760X.

Lannelongue, Loïc, Jason Grealey, and Michael Inouye (June 2021). "Green Algorithms: Quantifying the Carbon Footprint of Computation". In: *Advanced Science* 8.12, p. 2100707. ISSN: 2198-3844, 2198-3844.

Love, Michael I, Wolfgang Huber, and Simon Anders (Dec. 2014). "Moderated estimation of fold change and dispersion for RNA-seq data with DESeq2". In: *Genome Biology* 15.12, p. 550. ISSN: 1474-760X.

Setty, Manu et al. (Apr. 2019). "Characterization of cell fate probabilities in single-cell data with Palantir". In: *Nature Biotechnology* 37.4, pp. 451–460. ISSN: 1087-0156, 1546-1696.

Sing, T. et al. (Oct. 15, 2005). "ROCR: visualizing classifier performance in R". In: *Bioinformatics* 21.20, pp. 3940–3941. ISSN: 1367-4803, 1460-2059.

Soneson, Charlotte and Mark D Robinson (Apr. 2016). "iCOBRA: open, reproducible, standardized and live method benchmarking". In: *Nature Methods* 13.4, pp. 283–283. ISSN: 1548-7091, 1548-7105.

Zappia, Luke, Belinda Phipson, and Alicia Oshlack (Dec. 2017). "Splatter: simulation of single-cell RNA sequencing data". In: *Genome Biology* 18.1, p. 174. ISSN: 1474-760X.
